# Impact of light pollution on fishes: Mechanisms and ecological consequences

**DOI:** 10.1111/jfb.70656

**Published:** 2026-09-17

**Authors:** Hanxi Chen, Ulrika Candolin

**Affiliations:** ^1^ Department of Organismal and Evolutionary Biology University of Helsinki Helsinki Finland

**Keywords:** Anthropogenic stressor, ecological interactions, environmental change, evidence synthesis, illumination, visibility

## Abstract

The increasing prevalence of artificial light at night (ALAN) is of growing ecological concern. By altering natural light regimes, ALAN affects the behaviour, physiology and life‐history of organisms. While research has largely focused on terrestrial ecosystems, aquatic ecosystems are increasingly exposed. Here, we systematically review current knowledge of the impacts of ALAN on fishes and the underlying mechanisms and ecological consequences. We first discuss the factors influencing fish exposure to ALAN and how light can exert both direct and indirect effects. We then examine the potential consequences of these responses for ecosystem structure and functions, considering interactions with other environmental stressors, such as warming, noise and chemical pollution. To assess the current state of knowledge, we systematically review the literature. We find that most studies have focussed on the direct effects of ALAN on fish behaviour and physiology, whereas the fitness consequences of these responses and the impacts on population dynamics, species interactions and community composition remain largely unexplored. Notably, no study has assessed the broader implications for ecosystem functions. We identify key research priorities to advance understanding of both the short‐ and long‐term effects of ALAN on fishes and to inform effective mitigation strategies. In particular, more research is needed on the ecological consequences of fish responses to ALAN. We conclude by discussing potential solutions to light pollution, emphasising the importance of integrating economic, social and environmental considerations in the development of environmentally friendly lighting practices.

## 1 INTRODUCTION

The use of artificial light at night (ALAN) has increased exponentially within the last century and is expected to further increase in parallel with global human population growth (Kyba et al., 2023). This places light pollution as one of the fastest expanding environmental issues. It alters the natural light regimens that organisms have adapted to over millions of years, including daily, lunar and seasonal cycles of light, as well as visibility and habitat characteristics (Gaston & Sanchez de Miguel, 2022). The changes influence the behaviour, physiology and life‐history of organisms, with potential fitness consequences (Candolin & Filippini, 2025; Dominoni et al., 2016; Gaston et al., 2017). The increased use of broad‐spectrum light‐emitting diodes (LEDs) has further worsened the situation, as these emit more blue light than older lamps and consequently have a stronger effect on physiology than older light sources (Gaston & Sanchez de Miguel, 2022).

Aquatic ecosystems are increasingly exposed to light pollution at night, ALAN (Bassi et al., 2022; Elvidge et al., 2025; Hölker et al., 2023; Marangoni et al., 2022; Miller & Rice, 2023; Zapata et al., 2019). Especially freshwaters and coastal ecosystems are exposed due to their proximity to human activity and infrastructure, but also open waters because of light from shipping fairways, fisheries, and oil and gas rigs. Waters further from the light sources can be affected through skyglow, when light emitted upwards from strongly illuminated areas is reflected to earth by particles in the air, illuminating areas far from the light source (Davies et al., 2014; Smyth et al., 2022).

Artificial night light intensities at water surfaces can reach up to 200 lux, such as in harbours (Bolton et al., 2017). This is magnitudes higher than natural night light levels that reach a maximum of 0.05–0.2 lux during full moon and clear sky (Kyba et al., 2017). The increased exposure is of particular concern in shallow waters that are often highly productive and serve as nurseries for many species and support a significant part of biodiversity (Häder & Gao, 2023). Fishes are key components of many aquatic ecosystems that regulate the abundance and biomass of other species and mediate the flow of energy and matter through the food web (Villéger et al., 2017). Changes in their traits, such as in their behaviour, distribution and species interactions, can have far‐reaching ecological consequences (Candolin & Rahman, 2023). Most fishes inhabit the photic, lit zone (the uppermost layer of the water body) and, hence, are increasingly exposed to light pollution. Knowledge of how they respond to ALAN, and the consequences their responses can have for the ecosystem, is crucial for understanding the changes occurring in aquatic systems and for developing efficient management actions to prevent large‐scale ecological disruptions.

Here, we assess the state of knowledge regarding the impact of ALAN on fishes by systematically reviewing the literature and discussing the mechanisms underlying their response and the ecological consequences. We begin with explaining how the properties of aquatic ecosystems determine the light that reaches fishes and how the light can influence them both directly and indirectly. We discuss underlying mechanisms and consequences—from the individual to the ecosystem level—and how interactions with other human‐induced disturbances, such as climate change, noise and chemical pollution, can amplify the impact. To assess the current state of research, we perform a systematic search of the literature on fish responses to ALAN, the mechanisms behind the responses and the ecological consequences. We synthesise and analyse the findings and highlight research avenues in need of more attention for further progress within the field. We end with discussing how the negative effects of artificial light on fishes and aquatic ecosystems could be minimised.

## 2 DEPENDENCE ON ECOLOGICAL CONDITIONS, LIGHT QUALITY, FISH VISION AND TIMING

Light is attenuated with water depth, with the degree of attenuation depending on the physical properties of the water. In clear water, such as in the open ocean, blue wavelengths (around 475 nm) penetrate the deepest, even down to 1000 m, while in more turbid coastal and inland waters, the light shifts towards longer wavelengths, to green and red wavelengths, because of particles in the water that absorb the light in a wavelength‐dependent fashion (Mobley, 1994). The underwater lightscape is further determined by the topography of the habitat, such as by shading terrestrial plants and aquatic vegetation (Tidau et al., 2021).

Fishes adapted to different depths, habitats and activity times differ in their sensitivity to artificial light, both its intensity and spectrum. Vision is an important sensory system of most fishes, with the majority having colour vision, some perceiving ultraviolet or near‐infrared light and some even polarised light (Marshall et al., 2019; Musilova et al., 2021). Deep‐sea fishes can only sense the presence of light (scotopic vision) and are highly sensitive to even small amounts of light (Musilova et al., 2021). They can be profoundly influenced by light emitted from ships and from gas and oil rigs, as the light can extend below the photic zone, that is, beyond 200 m in clear ocean water (Berge et al., 2020). Similarly, fishes in polar waters, where there is no daylight for several months of the year, are likely to be sensitive to artificial light. A study in Arctic waters found fishes to avoid the illumination from a ship down to a depth of at least 200 m as well as 200 m from the ship (Berge et al., 2020). Nocturnal fishes prioritise light detection over colour detection by having rod dominated retinas and can be affected by even very weak levels of ALAN as they have extremely sensitive scotopic visual anatomy (Fogg et al., 2023). Day active fishes may again prolong their daily activity time or experience increased stress if the light limits the time for rest.

The sensitivity of fishes to the spectrum of light is also habitat and species specific (Musilova et al., 2021). For instance, threespine stickleback *Gasterosteus aculeatus* adapted to lakes with more turbid water are more sensitive to light of longer wavelengths than those in clear water lakes, as longer wavelengths are attenuated less than shorter wavelengths in turbid compared to clear water (Rennison et al., 2016). This turbidity‐dependent sensitivity has resulted in the impact of ALAN on sexual maturation depending on the spectrum of the light in algae‐turbid waters, with yellow light of longer wavelengths advancing sexual maturation more than white light of shorter wavelengths (Bäckroos et al., 2026). Differences in sensitivity to the spectrum of light can also influence movements and habitat choice of fishes. For instance, the cyprinid *Ptychobarbus kaznakovi* avoids areas illuminated by yellow and red light more than areas illuminated by green and blue light (Lin et al., 2021).

Species that depend on the lunar cycle for the timing of activities, such as for synchronised spawning and larval recruitment, or for behaviours related to tides, such as migration, can be affected by even weak nocturnal illumination that masks natural changes in moonlight (Fukunaga & Takemura, 2026). For instance, the settlement by larval coral reef fishes is desynchronised from the full moon—which it under undisturbed conditions depends on—by light pollution (Schligler et al., 2026).

Temporal characteristics of ALAN, timing and duration of exposure, are also critical determinants of responses of fishes to the light, as circadian rhythms and seasonal adaptations influence their physiological and behavioural responses (Liu et al., 2022). For instance, many fishes are more sensitive to ALAN during the reproductive period when light conditions influence hormone production (Brüning et al., 2018; Hölker et al., 2023). Shorter exposures to ALAN, during fewer nights rather than over extended periods, have less impact on fishes (Weschke et al., 2024), as may restricting the nightly period of illumination to the early part of the night or by using motion detectors that limit the duration of artificial illumination (Elgert et al., 2021).

## 3 DIRECT EFFECTS OF ALAN

Exposure to ALAN, whether through direct exposure or sky glow, can influence fishes, especially their physiology, behaviour and life‐history, as well as their reproductive success and survival. The impact depends on their sensory system (how they receive and process the light), the evolved reaction norms to the light (such as avoidance or attraction) and their habitat choice. Research in aquaculture has especially enlightened us on the direct impact that light conditions—such as photoperiod and light quality—can have on fish physiology and behaviour (e.g., Boeuf & Le Bail, 1999; Ma et al., 2026).

### 3.1 Physiological and developmental responses

ALAN can influence the physiology of fishes through effects on hormones and neuropeptides. Even very low levels of light, under 1 lux, can decrease the production of the pineal hormone melatonin that regulates the endogenous clock (Brüning et al., 2018; Grubisic et al., 2019; Kupprat et al., 2020). ALAN can also reduce the production of thyroid hormones that regulate metabolism, development and activity (Kupprat et al., 2021; O'Connor et al., 2019). The impact of ALAN on the stress hormone cortisol is more equivocal, as most studies on fishes find no effect or a weak effect (Brüning et al., 2015; Newman et al., 2015; Szekeres et al., 2017).

ALAN is well known to influence sex hormones (steroids) and thereby reproduction (Kloas et al., 2024). This is exploited in aquaculture where light conditions are manipulated to alter gamete and filet production (Norberg et al., 2004). In wild fishes, ALAN can similarly influence the concentrations of sex hormones (Brüning et al., 2018). In addition, the spectrum of the light can have an impact. For instance, yellow light advances sexual maturation more than white light in threespine stickleback (Bäckroos et al., 2026).

The physiology and development of early life stages are often sensitive to ALAN. The light can increase metabolic demands of embryos (Roost et al., 2025), reduce hatching success (Fobert et al., 2021) and influence offspring viability after hatching (Lucon‐Xiccato et al., 2023).

The impact of ALAN on the expression of genes has so far received little attention in fishes. However, research studies on other species show that altered gene expression is a common pathway behind impacts of ALAN (Alaasam et al., 2024). Some evidence has recently emerged for fishes for effects of fluorescent light and 24 h of constant light (Boswell et al., 2019; Zhang et al., 2024).

ALAN could induce transgenerational changes via compounds transferred in eggs and sperm, and by influencing the care behaviour of parents. While evidence for transgenerational effects in fishes is mounting for other environmental disturbances (Abdelnour et al., 2024), the impact of light pollution is poorly known. A recent study found the exposure of adult zebrafish to ALAN to reduce the movements of their offspring (Li et al., 2024).

### 3.2 Behavioural and phenological responses

The first visible response of individuals to ALAN is often behavioural (Tuomainen & Candolin, 2011; Wong & Candolin, 2015). This can be the attraction to the light (Nelson et al., 2022; Tabor et al., 2021; Weschke et al., 2024) or its avoidance (Geoffroy et al., 2021). For instance, illumination of an estuary in South Africa attracted fish and increased their abundance (Becker et al., 2013). ALAN can also increase the activity of fishes, as noted for several diurnal species (Georgiou et al., 2024; Pulgar et al., 2019). Increased activity can be costly in augmenting energy expenditure and reducing time for sleep (Pulgar et al., 2019), but could improve foraging success (Czarnecka et al., 2019; Tałanda et al., 2022). ALAN may also alter the diet, as found around fishing lights where fishes increase feeding on prey attracted by the light, such as smaller fishes (Tsounis & Kehayias, 2021).

Reproductive behaviour can be altered by underlying physiological changes or be directly influenced by ALAN, such as choice of spawning site (Closs et al., 2023; Kloas et al., 2024). ALAN can also interfere with the optimal timing of activities, such as reproduction and migration (Riley et al., 2015), influence navigation and the ability of fishes to orient in space (Pulgar et al., 2023) and impair learning (Lucon‐Xiccato et al., 2023).

## 4 INDIRECT EFFECTS OF ALAN

Indirect effects arise when ALAN influences the abiotic (other than light conditions) or biotic environment and this, in turn, influences the species. For instance, ALAN may reduce the availability of suitable prey if these avoid the light or are less active, or the light may increase predation risk by improving the efficiency of predators. Research on other stressors indicates that indirect effects can be larger than direct effects, especially through effects on species interactions (Cahill et al., 2013). Species interactions are the cornerstones of ecosystems and changes in them—because of changes in the behaviour or abundance of some species—can have knock‐on effects on other species and cause a cascade of effects (Rahman & Candolin, 2022).

While evidence for indirect effects of ALAN on species is increasing across taxa (Hirt et al., 2023), relatively little is known about effects on fishes. ALAN can alter shoaling behaviour and risk taking, which could influence predation rate (Becker et al., 2013; Kurvers et al., 2018). Changes in food availability, such as prey abundance or distribution, could similarly influence species interactions. For instance, ALAN alters the diel vertical migration behaviour of zooplankton (Lehtonen & Candolin, 2025), the emergence of aquatic insects from waters and their return to oviposit, and suppresses drift of invertebrates in running water (Manfrin et al., 2017; Perkin et al., 2014). Furthermore, ALAN could influence fishes through effects on primary production (Maggi et al., 2020).

Attraction of parasites, or their intermediate hosts, to artificial light could further influence fishes. This has been found in fish farms where illuminated cages attract cestode‐infested copepods, which then transmit the parasite to the farmed fishes (Sichrowsky et al., 2013). Changes in habitat choice of fishes could further influence exposure to parasites. Atlantic salmon, for instance, position themselves closer to the surface when exposed to ALAN, which increases their exposure to lice infestations (Hevroy et al., 2003).

## 5 IMPACT ON POPULATIONS

Changes in the traits of individuals or in their reproductive success and survival can influence the size, structure and distribution of populations. However, while an increasing number of studies find ALAN to influence these characteristics (reviewed in Bassi et al., 2022; Ferretti et al., 2025; Marangoni et al., 2022; Zapata et al., 2019), the impact on population dynamics is poorly known. Indirect evidence suggests that population‐wide effects could be common. For instance, exposure to ALAN hampers sexual maturation and reproductive behaviours of many fishes (Brüning et al., 2018; Fobert et al., 2021; Foster et al., 2016) and can decrease the growth and survival of juveniles and alter their distribution (Brüning et al., 2011; Li et al., 2024; Lucon‐Xiccato et al., 2023; O'Connor et al., 2019; Schligler et al., 2021). Altered predation risk or food intake of adults could further influence population dynamics, as could effects on their body condition because of the stress induced by artificial light or increased parasite infections (MacAulay & Cable, 2024).

## 6 IMPACT ON COMMUNITY AND ECOSYSTEM

Changes in the traits and population dynamics of fishes, as well as in their interactions with other species, can influence the structure of ecosystems—the biodiversity and biomass of organisms at different trophic levels—as well as their functions, such as nutrient cycling, biomass turnover and carbon sequestering. Such changes can, in turn, influence the stability of the ecosystems and resilience to further disturbances, including other stressors, such as global warming, chemical pollution and noise (Candolin & Rahman, 2023).

Evidence for effects of ALAN on the structure of fish communities is growing. Waters exposed to light pollution often harbour higher abundances of fishes (Barker & Cowan, 2018; Becker et al., 2013; Bolton et al., 2017; Nelson et al., 2022) and altered species composition (Weschke et al., 2024). The light can attract fishes directly or through increased prey abundance (Bolton et al., 2017; Ganguly & Candolin, 2023; Tałanda et al., 2022). Changes in the composition of fishes can have cascading effects on the entire species community through bottom‐up, top‐down and lateral effects (Hirt et al., 2023; Hölker et al., 2023; Perkin et al., 2014). Yet, little is currently known about cascading effects caused by ALAN or their impact on ecosystem processes. Changes in their diversity, abundance, behaviour and interactions could have profound effects on ecological processes, including the resilience of ecosystems towards other disturbances and ecosystem services, such as food provisioning, clean water and pest control (Candolin, 2019; Villéger et al., 2017).

Habitats that are not illuminated at night can be indirectly influenced by ALAN, through effects on connected habitats. For instance, altered migration of fishes between open water and illuminated shallow coastal waters or running waters could influence the flux of matter and energy between these habitats. Even terrestrial habitats could be influenced if changes in fish communities influence their terrestrial predators, such as birds and mammals, or the emergence of aquatic insects from the water (Manfrin et al., 2017).

## 7 INTERACTIONS WITH OTHER HUMAN‐INDUCED DISTURBANCES

Humans are exposing aquatic ecosystems to a multitude of stressors, from climate change and habitat modification to various forms of pollution, such as noise, chemical contamination and nutrient addition. How these stressors combine to influence fishes and their ecological functions is poorly known.

Interactive effects are likely between ALAN and temperature, given that both can be used as cues for circadian and circannual cycles (Bradshaw & Holzapfel, 2010). If the cues are mismatched, maladaptive responses may follow. For instance, the combination of cool winter temperature with long summer photoperiod altered thyroid levels in the mosquitofish *Gambusia holbrook*i, which reduced locomotor performance, metabolic rate and mitochondrial efficiency (Le Roy & Seebacher, 2020).

Interactive effects between light pollution and noise have been recorded for other taxa than fish, especially for birds, ranging from antagonistic to synergistic and emergent effects (Halfwerk & Jerem, 2021). Studies on fishes are largely lacking, but a study on zebrafish detected no interaction between artificial light and noise, although both stressors separately influenced activity and habitat choice (Shafiei‐Sabet et al., 2016).

Chemical pollution is a growing environmental issue that similarly to ALAN can influence biological rhythms and, hence, could interact with light pollution (Thoré et al., 2024). In support of this, the combination of ALAN with a flame retardant (hexabromocyclododecane) synergistically disrupted vision and sleep in zebrafish larvae (Cao et al., 2024). Nutrient addition, eutrophication, is especially likely to interact with ALAN, as it alters the penetration of light in water (Mobley, 1994). Similarly, eutrophication‐induced changes in pH and dissolved oxygen levels could influence the physiology of fishes and thereby interact with ALAN, as could the increasing amount of microplastics in water.

Human constructions that alter habitats, such as seawalls, breakwaters, canals, dams and hydroelectric installations, could interact with ALAN by altering the distribution of light within the habitat (Ferretti et al., 2025). This could influence predation risk, foraging success, habitat choice and the distribution of fishes.

Invasions of non‐native species are a further human‐induced issue that could interact with ALAN, especially through altered species interactions. Invading species that benefit from improved visibility under ALAN could reduce the abundance of native fishes or alter their distribution through increased predation or intensified competition for resources. Alternatively, invaders could form new prey species for native fishes, in which case ALAN could promote predation on them. Yet, despite the steady increase in species invasions, the prevalence of such interactive effects is unknown.

## 8 SYSTEMATIC REVIEW OF THE LITERATURE

To assess the research that has been performed on responses of fishes to ALAN and the consequences their responses can have from the population to the ecosystem level, we followed the guidelines of the Collaboration for Environmental Evidence (CEE) http://www.environmentalevidence.org/information-for-authors, which complies with Reporting Standards for Systematic Evidence Synthesis (ROSES) (Haddaway et al., 2018). Our inclusion and exclusion criteria for including a study were as follows:

1. Test for effects of artificial light at night on individual, population or community characteristics of fishes, excluding constant day and night light, night light over 200 lx at water surface and light submerged under the epipelagic zone, under 200 m.

2. Have a control, such as a before/after study or paired sites/treatments, or use different light intensities, spectrum or time exposure.

3. Exclude studies that use artificial light to improve the efficiency of fishing gear, such as in by‐catch studies, and studies using submerged lights to alter swimming depth of farmed, enclosed fish, as these studies, which are numerous, do not reflect the response of fishes to the main forms of ALAN and could bias the results to more uncommon, specific situations.To develop the search string and evaluate its sensitivity, we collected 41 benchmark studies from different sections of this review to make it as broad as possible (see benchmark studies in Appendix S2 in the Supporting Information). We used the workflow described by Lagisz et al. (2025) and searched for the benchmark studies in Web of Science Core Collection, using their DOI numbers and the Boolean operator ‘OR’. We found all 41 studies and iteratively developed a target search string until all benchmark studies were included and our search sensitivity (search string evaluation) was 100%. The final target search string was:

(‘artificial night light*’ OR ‘night‐time light*’ OR ‘nocturnal illumination’ OR ‘outdoor light*’ OR ‘artificial light’ OR ‘light* at night*’ OR ‘anthropogen* light*’ OR ‘urban light*’ OR ‘light pollution*’ OR ‘nightlight*’ OR ‘man made light*’ OR ‘part night light*’ OR ‘street light*’ OR streetlight* OR ‘vertical light beam*’ OR ‘street lamp*’ OR streetlamp* OR ‘sky glow*’ OR skyglow*) AND (fish* OR zebrafish OR salmon).

We used the target search string to search three databases: Web of Science Core Collection, Scopus and PubMed (date of search 10 March 2025). We used the software Rayyan (https://www.rayyan.ai/) to upload the studies, and after the deletion of duplicates, were left with 809 publications. We screened these for the inclusion/exclusion criteria, which resulted in 131 publications after title and abstract screening. The agreement rate between the two reviewers (HC and UC) was 100% for the first 150 publications (out of 809) after discussions of divergence or uncertainty in ratings. After full‐text screening, we were left with 55 publications with 100% agreement between the reviewers after discussions of divergence or uncertainty in ratings. From these studies, we noted light characteristics during days and nights, duration of ALAN treatment and categorised studies according to:

1. Habitat (marine, freshwater, anadromous, catadromous), microhabitat (coastal, pelagic, reefs, lentic, lotic), life stage (egg, juvenile, adult) and sex of fish.
2. Type of study (field, laboratory, mesocosm) and study design (field observation, field experiment, lab experiment, cage experiment, mesocosm experiment).
3. If direct or indirect effects, or both, were investigated.
4. Responses recorded (abundance, behaviour, cognition, distribution, gene expression, growth, physiology, reproduction, survival), and if there was a significant effect on the recorded response.
5. If the response was adaptive, maladaptive, or if adaptedness was unknown in relation to fitness‐related traits (survival, growth, reproduction).
6. The ecological level at which effects were recorded: individual, population, species interaction, community or ecosystem.

Below we summarise the results from the systematic review.

### 8.1 Study characteristics

In total, 29 different species and 6 fish assemblages were included, 141 measures of responses to ALAN, and 248 measures of correlations between light intensity and response (multiple light intensities were used in some studies). Freshwater fishes have received most attention, with slightly more research on lentic (standing water) than lotic (flowing water) species (Figure 1). Research studies on marine fishes have concentrated on coastal and reef habitats and less on pelagic fishes (Figure 1).

**FIGURE 1:**
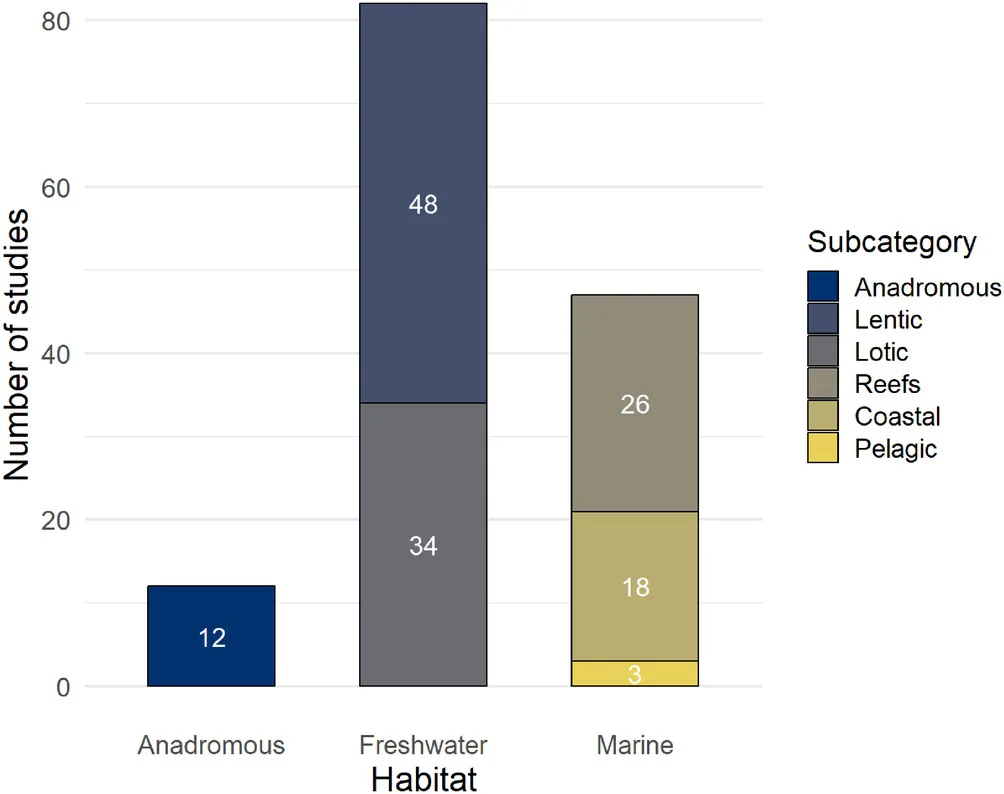
Number of studies on effects of ALAN on fishes in different habitats (*N* = 141).

Laboratory studies dominate over field studies, and most field studies are experimental, few observational (correlative) (Figure 2). Both adults and juveniles have been investigated, but few have considered the egg stage (Figure 3). Several studies do not mention the stage or age of the fish, but assumedly most of these were on adult fish (Figure 3). Most studies do not separate between males and females, and there is no clear bias in the use of the sexes (Figure 4). An overview of the relationship between these studies is presented in Figure 5.

**FIGURE 2:**
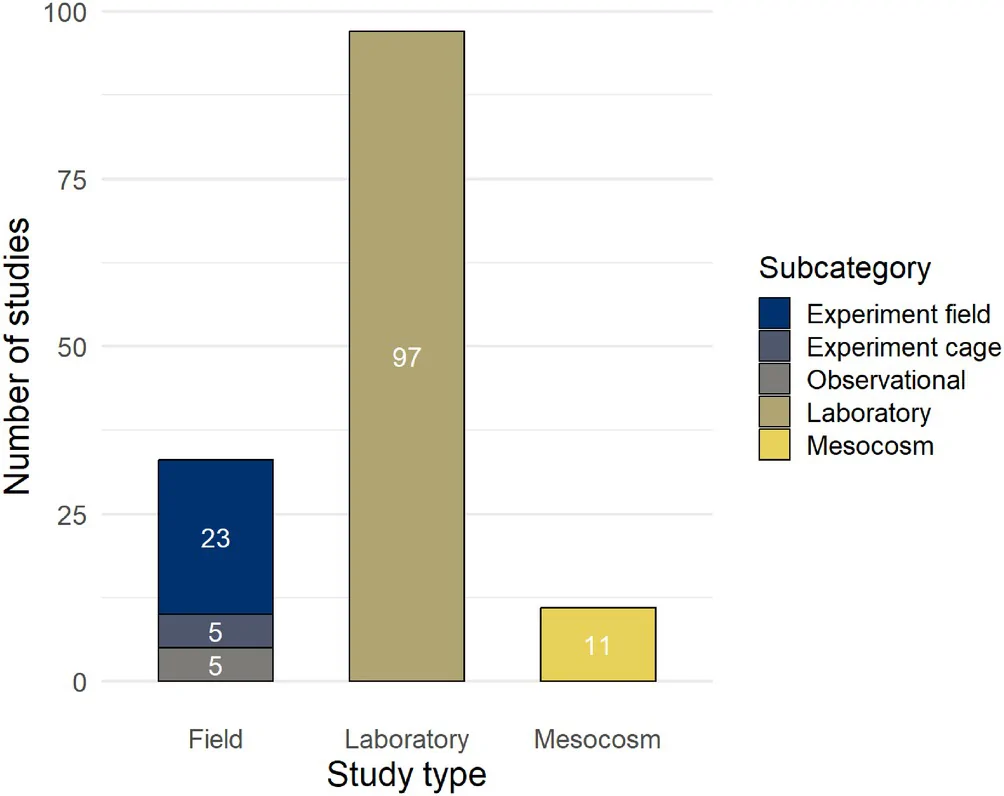
Number of studies on effects of ALAN on fishes using different research approaches (*N* = 141).

**FIGURE 3:**
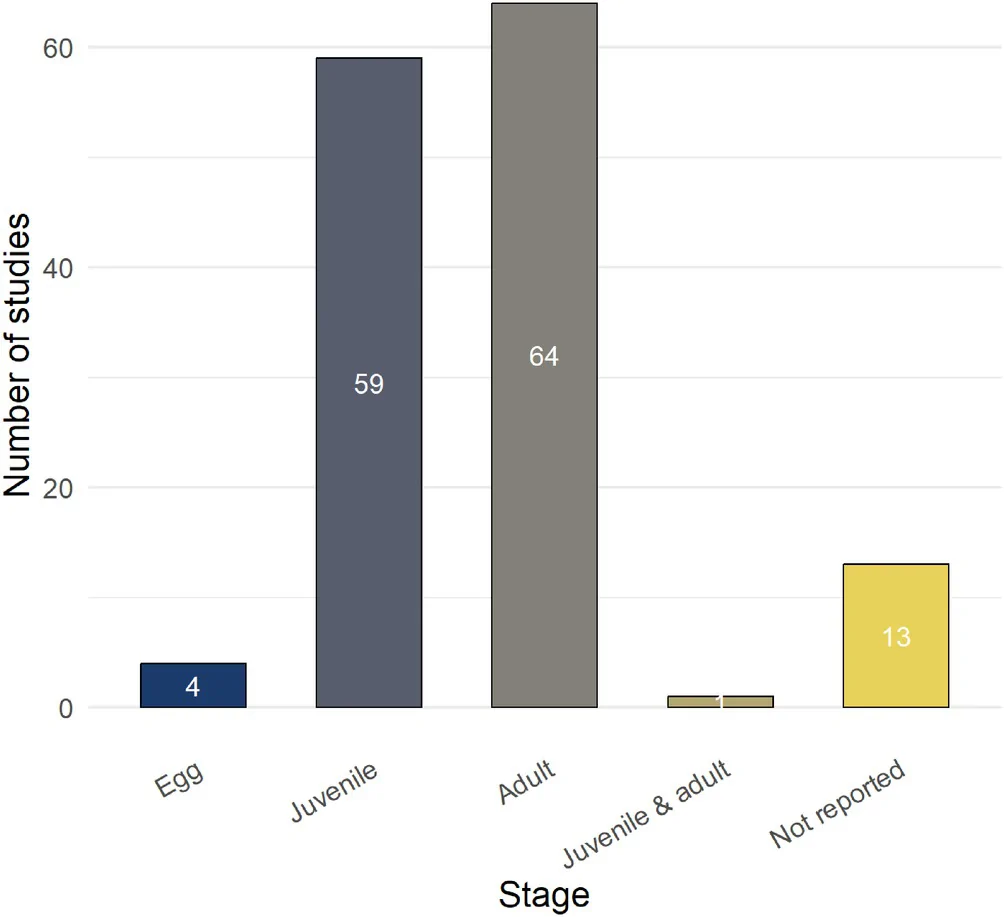
Number of studies on effects of ALAN on fishes using different life stages (*N* = 141).

**FIGURE 4:**
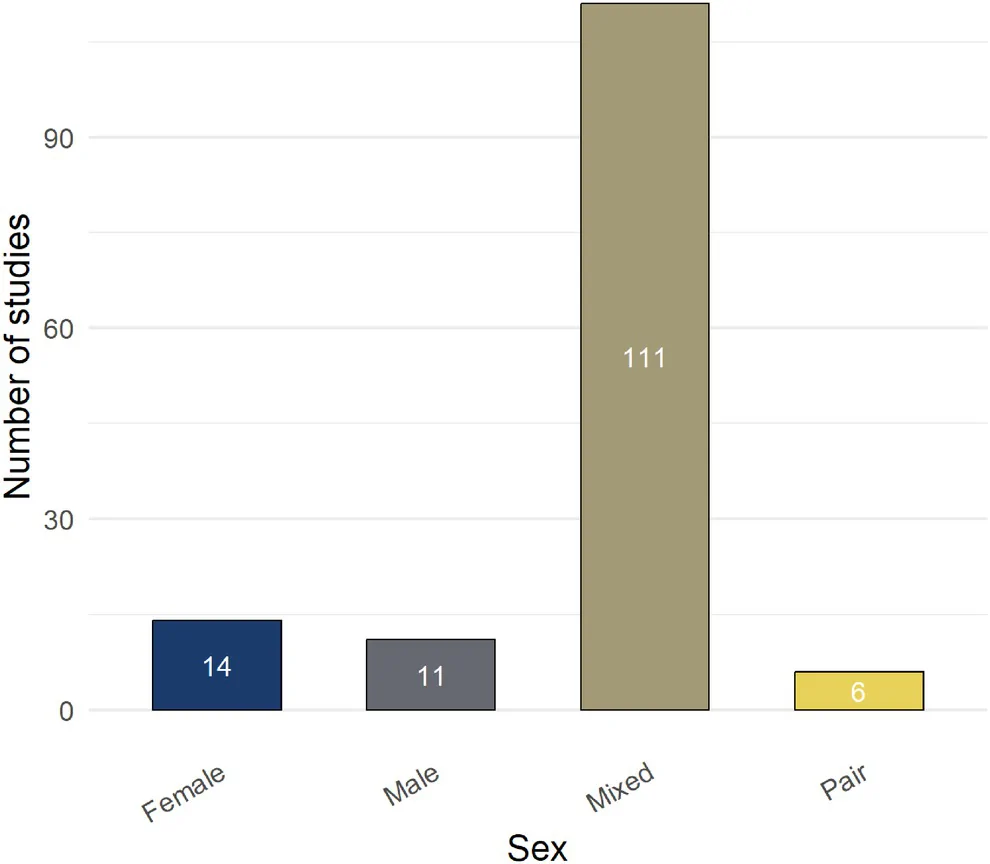
Number of studies on effects of ALAN on fishes in relation to sex (*N* = 141).

**FIGURE 5:**
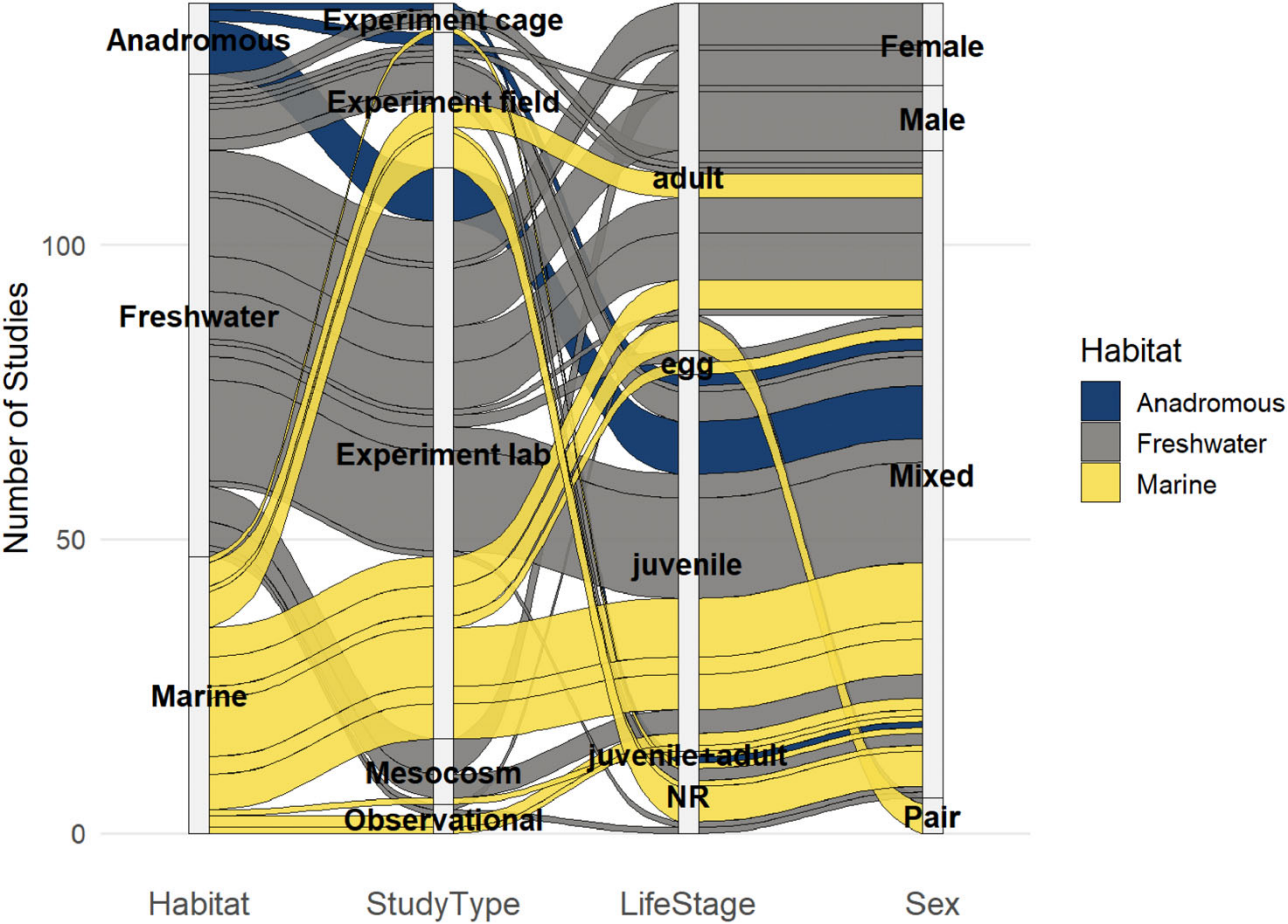
Alluvial diagram of studies on effects of ALAN on fishes based on habitat, study type, life stage and sex (*N* = 141).

### 8.2 Targets and effects

All studies but one investigated direct effect of ALAN on fishes, and the single study on indirect effects assessed effects of predator presence on juvenile survival. Behavioural studies dominated, followed by physiological recordings (Figure 6, number of studies are presented in Appendix S1, Table S1). The majority of studies, 61%, found an effect of ALAN on recorded responses (Figure 6).

**FIGURE 6:**
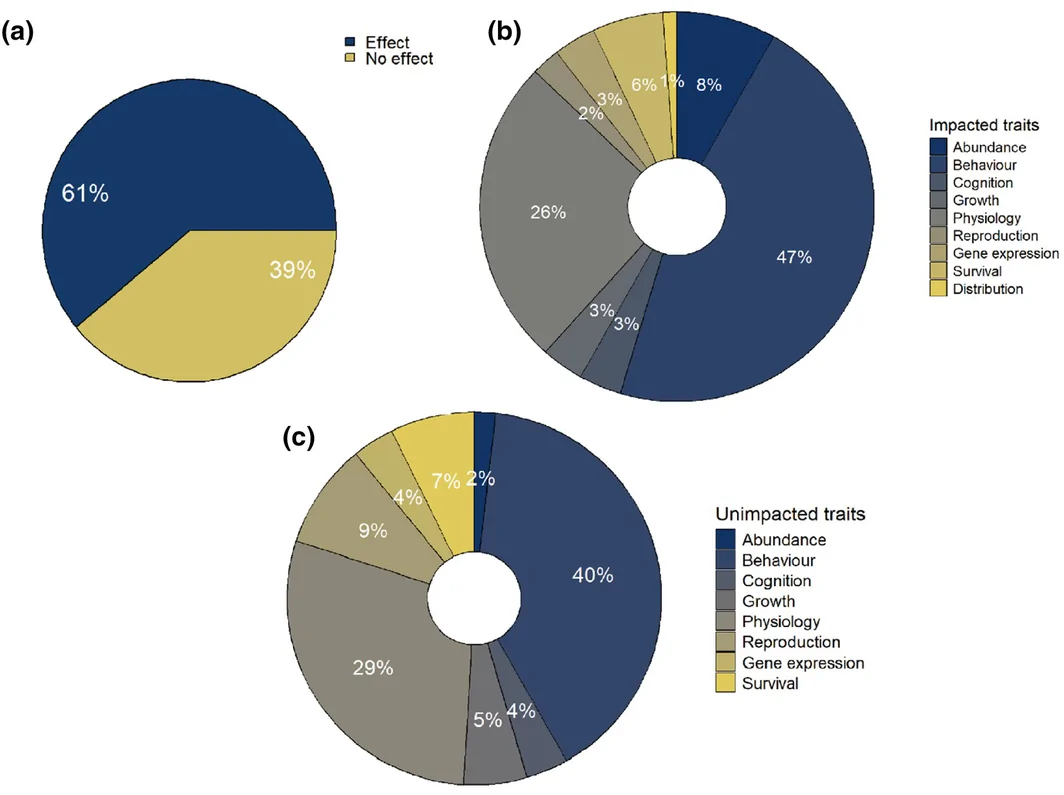
(a) Proportion of studies that found an effect, or did not find an effect, of ALAN on fishes (*N* = 141), (b) the proportional distribution of responses that were affected (*N* = 86) and (c) the proportional distribution of responses that were not affected (*N* = 55).

Whether the responses to ALAN were adaptive in improving survival, growth or reproductive success was seldom assessed, and those that did assess (*N* = 22) reported more often maladaptive (*N* = 8) than adaptive (*N* = 2) responses, while most studies (*N* = 12) found no effect on survival, growth or reproductive success (Figure 7).

**FIGURE 7:**
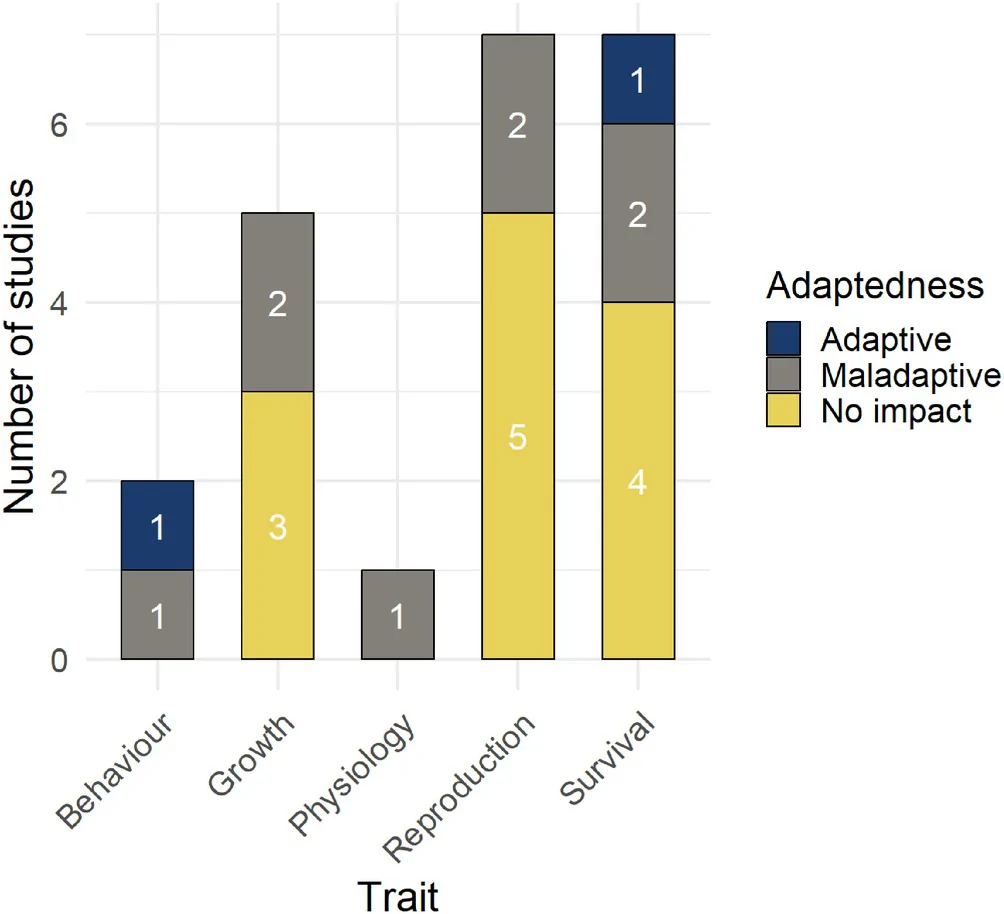
Number of studies that assessed the adaptedness of responses of fishes to ALAN in improving survival, growth or reproduction, with the colours indicating whether the response was adaptive, maladaptive, or had no impact. Numbers under bars show the number of studies investigating each response.

Effects at the population level were seldom assessed (Figure 8). Population size was most often assessed, in 10 out of 141 studies, with 3 of these finding ALAN to increase population size. Similarly, impact on species interactions was seldom assessed, and those that did assess focused on consumer‐resource interactions (foraging or predation) (Figure 8). Consequences for biodiversity, the composition of fishes and other species in the community were similarly seldom investigated, with none reporting decreases in biodiversity and most finding changes in composition (Figure 8). None of the included studies assessed effects on ecological processes such as nutrient cycling or the transfer of energy and matter through the food web.

**FIGURE 8:**
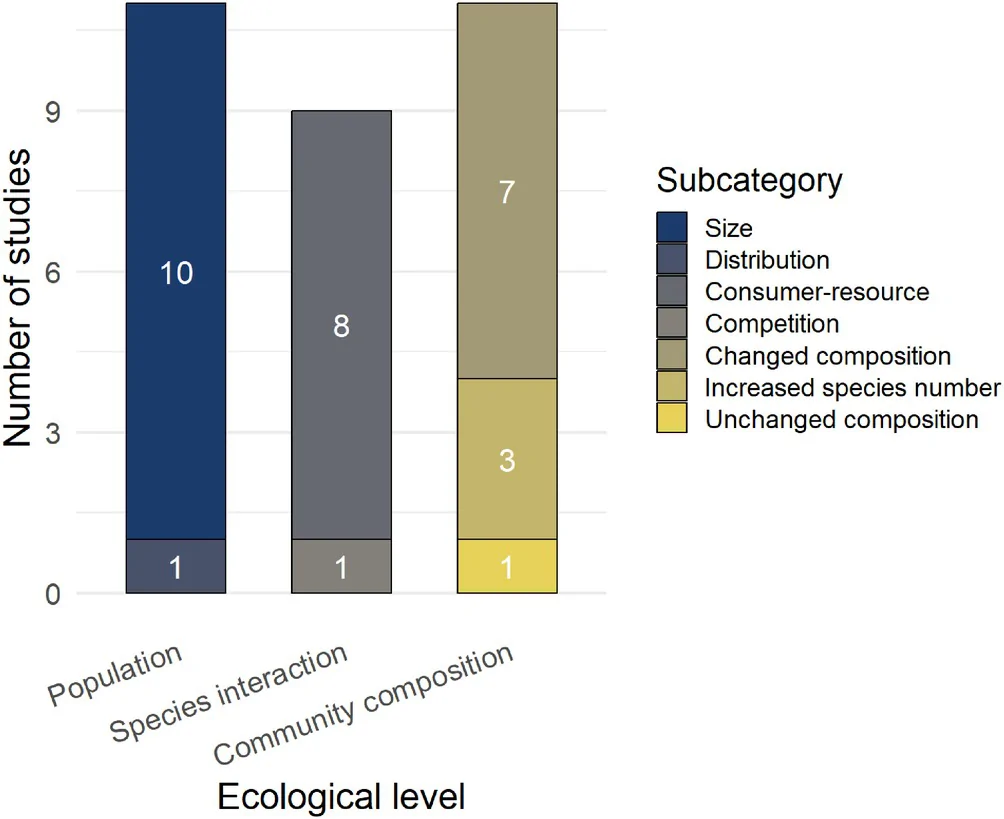
Number of studies investigating effects of ALAN on population characteristics, species interactions and community composition of fishes.

### 8.3 Light characteristics

Light intensity used in the studies did not influence the probability of finding an effect of ALAN, when we restricted the analysis to the 230 studies that reported intensities in lux (generalised linear mixed model [GLMM] with binomial error distribution, with impact or no impact of ALAN as binary response variable, light intensity as fixed factor and study identity as random factor: *F*_1,228_ = 0.06, *p* = 0.81, Figure 9). The pattern did not significantly differ among response categories (adding response categories to the GLMM model, with cognition, growth and gene expression merged with physiology and abundance deleted given low sample size (*N* = 5); interaction between intensity and response category: *F*_3,221_ = 0.20, *p* = 0.65, Figure 9, see Appendix S1, Figure S1 for logistic regression curves).

**FIGURE 9:**
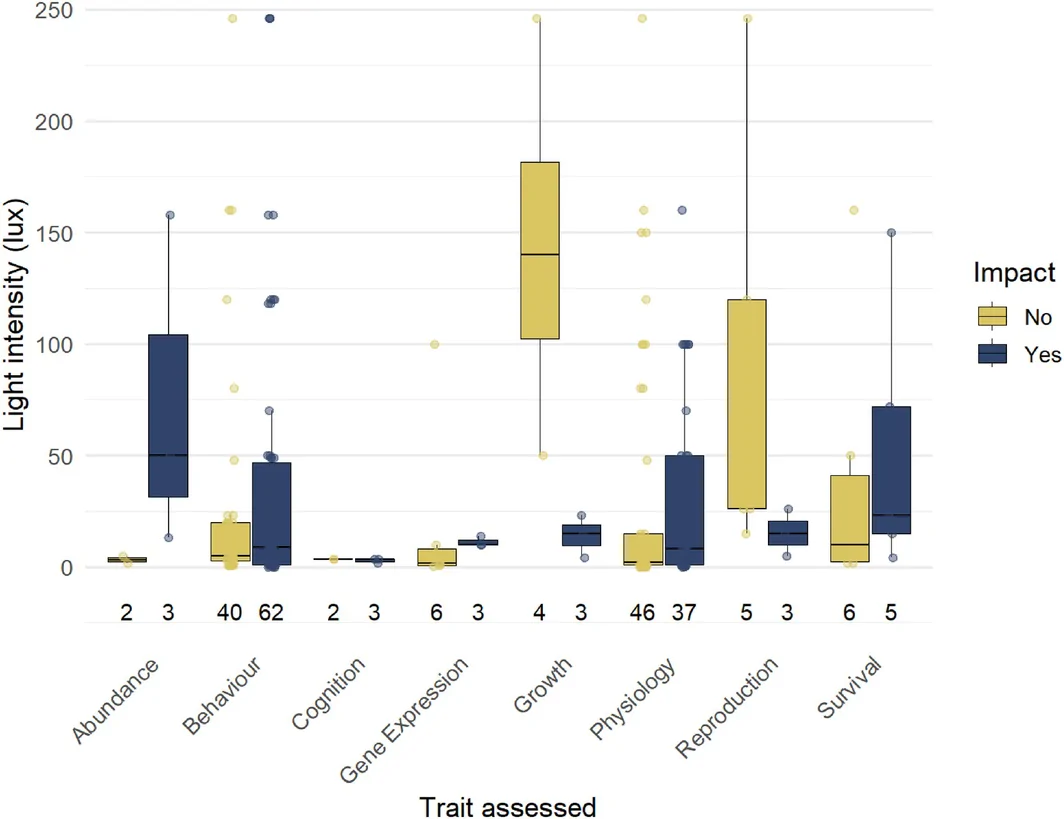
Light intensities used in studies that reported these in lux and found, or did not find, an impact of ALAN on fishes. Number of recordings is given under columns (*N* = 230).

The colour of the light was noted for 69 of the 141 recordings, with most using white light (Figure 10, see distribution among response categories in Appendix S1, Figure S2). Other common colours were red and green, but given the low number of studies using these colours, no clear conclusions can be drawn about the impact of the light spectrum. Most studies reporting an effect of ALAN did not specify the colour or spectrum of the light (Figure 10).

**FIGURE 10:**
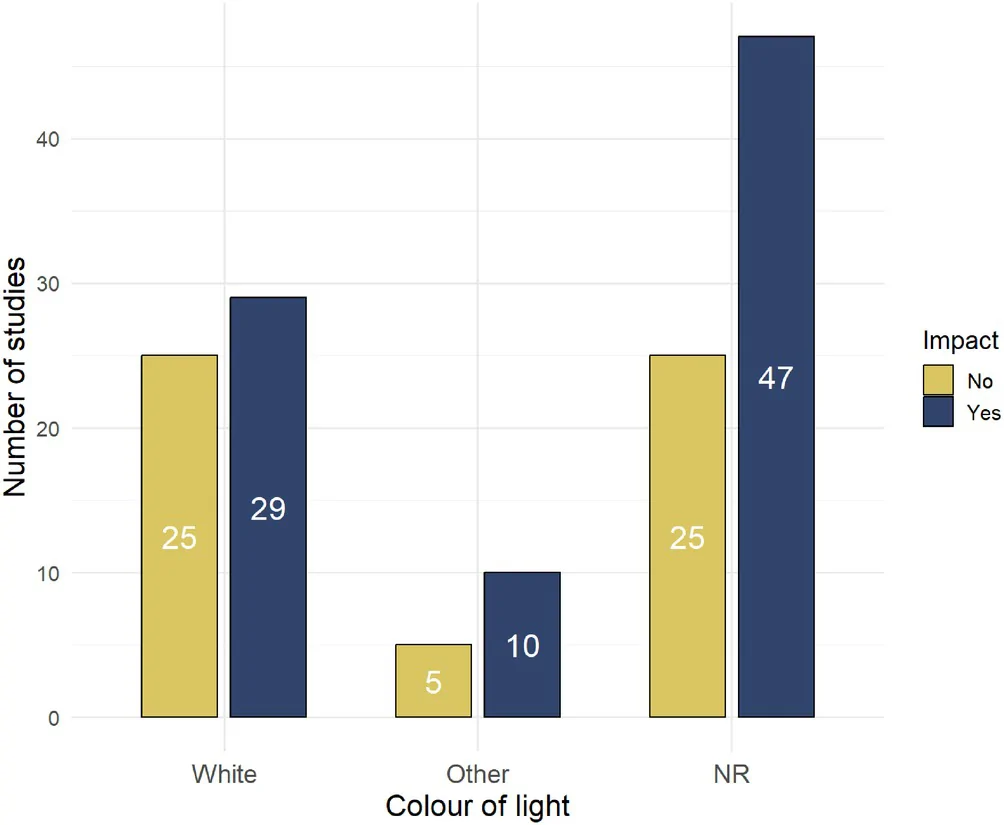
Number of studies that found or did not find an impact of ALAN on fishes depending the colour of the light; white, other than white, or not reported (*N* = 141).

The duration of ALAN varied from flashes of light from passing cars to up to 2 years of exposure. The duration exhibited a weak tendency to affect the probability of detecting an effect of ALAN, with shorter durations tending to be associated with a higher probability of effect (GLMM with binomial error distribution, with impact or no impact of ALAN as binary response variable, duration of exposure as fixed factor and study identity as random factor: *F*_1,246_ = 3.82, *p* = 0.052, Figure 11). The effect of duration differed among response categories, with behaviour being less affected than cognition, gene expression, growth and physiology (adding response categories to the model, with cognition, growth, gene expression and physiology merged into one group, while distribution (*N* = 1) and abundance (*N* = 9) were deleted given low sample sizes; interaction between duration and response category: *F*_3,234_ = 4.60, *p* = 0.033, Table 1, logistic regression curves are presented in Appendix S1, Figure S3).

**FIGURE 11:**
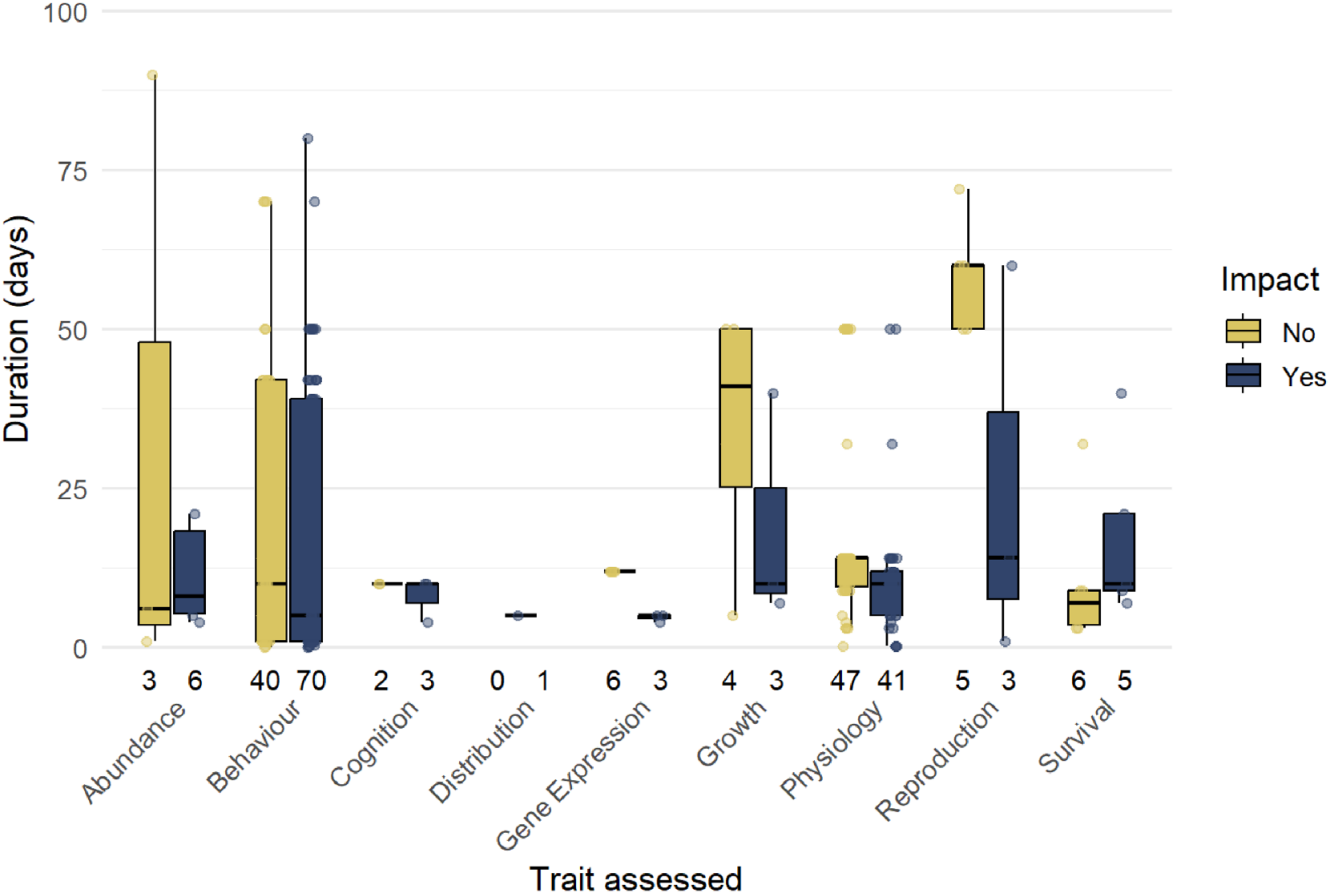
The duration of the exposure to ALAN in studies that found, or did not find, an impact on fishes. Number of recordings given under columns (*N* = 248).

**TABLE 1:** Pairwise comparison of the influence of the duration of exposure to ALAN on the probability of an effect on fishes for different response categories (with distribution and abundance deleted given low sample sizes) (*N* = 238).

Trait 1	Trait 2	95% CI	Odds ratio	P
Behaviour	Survival	[0.108, 2.018]	0.479	0.330
Behaviour	Reproduction	[0.051, 1.886]	0.346	0.257
Cognition, gene expression, growth, physiology	Behaviour	[0.272, 0.861]	0.486	0.010
Cognition, gene expression, growth, physiology	Survival	[0.223, 4.128]	0.983	1.000
Cognition, gene expression, growth, physiology	Reproduction	[0.105, 3.858]	0.71	0.727
Survival	Reproduction	[0.156, 13.587]	1.365	1.000

## 9 STATE OF THE RESEARCH FIELD

There is mounting evidence that ALAN influences organisms and ecosystems, including aquatic ecosystems (Candolin & Filippini, 2025; Gaston & Sanchez de Miguel, 2022; Hölker et al., 2023; Sanders et al., 2021). However, our systematic review of the literature reveals that effects on fishes and the ecological consequences are poorly known. Research has focussed on the direct impacts of ALAN on individual fish species, especially on their behaviour and physiology, while indirect effects through species interactions and the fitness consequences of the responses are largely unknown. Most of the studies on direct effects of ALAN find the light to influence fishes, also weak intensities and short durations of ALAN, and the few studies that have assessed the adaptive value of the responses usually find these to be maladaptive. However, the ultimate effect of the responses on population dynamics and ecological conditions is almost completely unknown. Yet fishes are key species of aquatic ecosystems that regulate the abundance of other species and mediate the transfer of energy and matter through the food web (Villéger et al., 2017). Thus, more research is needed on the consequences of the responses of individual fish for their populations, communities and ecological conditions. In particular, more research is needed on effects on interactions among species, as these are the backbone of ecosystems that define their structure and functions; changes to species interactions could have profound ecological consequences (Martins et al., 2024).

Most research has also concentrated on the intensity of the light while the impact of the spectrum, which is known to vary with depth and water quality, is left unknown. Given that the spectrum of artificial illumination is currently rapidly changing with the development of LED technology, more attention needs to be devoted to the impact that light quality has on fishes in different habitats.

The review also reveals that more research is needed on the mechanisms behind the responses and the causes of differences among species in how they respond. Physiological changes often underlie behavioural responses, but ALAN can influence behaviour also directly, such as through the choice of habitat or altered activity time. Changes in behaviour can in turn influence physiology, with bidirectional linkages between the two. Yet little research has been devoted to the link between physiological and behavioural responses within a light pollution context. Furthermore, differences among life stages in sensitivity to ALAN need more attention, considering that many fishes reproduce in shallow water and early life stages may stay in these for extended periods before moving to deeper water. Impact on early development can have considerable consequences later in life (Burton & Metcalfe, 2014).

There is also less information on the impact of the activity time of fishes on their responses to ALAN. Do diurnal, crepuscular and nocturnal fishes respond differently? Diurnal fishes may extend their activity times, which could result in less time for rest and sleep, with negative effects on fitness. Nocturnal fishes could again experience higher predation risk or lower foraging success under ALAN, with negative effects on fitness. Crepuscular fishes, on the other hand, could benefit if the time with weak light is extended. However, to our knowledge, no study has systematically investigated the impact of the activity time of fishes on sensitivity to ALAN.

Another major gap in our knowledge is the lack of information on the longer‐term effects of ALAN and the ability of fishes to plastically adjust and genetically adapt to ALAN. In terrestrial habitats, comparisons between urban and rural habitats show that species differ in their ability to adjust and adapt to urban conditions, including light conditions (Candolin, 2024; Hopkins et al., 2018). Considering the importance of fishes in influencing ecosystem structure, function and stability, more attention should be given to evolutionary processes and longer‐term consequences of ALAN.

Our review also reveals that more research is needed on interactions between ALAN and other anthropogenic stressors, given that aquatic ecosystems are exposed to a multitude of stressors and interactions among these are frequently found for other systems (Bradshaw & Holzapfel, 2010; Halfwerk & Jerem, 2021). Interactions can be additive, synergistic or antagonistic and profoundly alter the impact of single stressors (Munson et al., 2024). Yet, worrying little is known about interactions between ALAN and other stressors in impacting fishes.

Related to the complexity of ecosystems and influencing factors, more research should be conducted in the field, under natural conditions. Most studies to date have been conducted in the laboratory under simplified conditions. Ideally, research should combine observational and experimental field studies that show patterns in nature with experimental laboratory studies that can control for confounding factors and untangle pathways and mechanisms. While the systematic assessment focused on studies of ALAN, evidence from other applications of artificial lighting, particularly in aquaculture and fisheries, can provide complementary insights into fish responses to altered light environments. Artificial lighting has been extensively studied in aquaculture, yet this body of evidence has been comparatively underused in assessing the potential ecological consequences of ALAN for wild fishes. Such studies can provide valuable mechanistic insights into responses to altered light intensity, spectrum and photoperiod, although differences in exposure levels, lighting regimes and environmental context need to be considered when extrapolating these findings to natural systems.

A further important line of research is the development of objective and standardised methods for measuring light, especially for aquatic habitats for which measurements are sparse given limitations in the technology of underwater light loggers (Hölker et al., 2023). Standardised methods would allow the comparison of effects of ALAN among studies, species and ecosystems. Currently, measurements vary both within and across disciplines, from single‐point to full light field (full‐sphere) measurements. Similarly, units vary from light intensities based on human vision, lux, to full‐spectrum irradiance (or radiance) measurements presented as energy (W/m2) or photon flux (photons/m2/s) per area. Presenting light conditions as energy or photon flux combined with the spectral distribution would improve our understanding of the light conditions that fishes experience, as measurements in lux are adjusted for human spectral sensitivity and may not reflect the illumination that animals experience.

Finally, more research is needed on how to mitigate the negative effects of ALAN on fishes and aquatic ecosystems. Humans depend on artificial light for utility and safety, but the development of lighting systems has traditionally not considered environmental effects. This is slowly changing, with new technologies allowing the development of more environmentally friendly lighting systems. In this research, environmental, social and economic aspects need to be considered.

## 10 SOLUTIONS

Light pollution is relatively easy to manage and it may leave little residual effects once removed, depending on how fast populations recover or return to the habitat and how fast ecosystem functions are restored. By restricting the spatiotemporal distribution of ALAN, lowering its intensity and selecting spectral compositions that are less disruptive to organisms, light conditions can become more environmentally friendly without compromising human safety and the utility of the light (Evans, 2023).

The spatial distribution of artificial light can be limited by restricting illumination to areas that specifically need illumination, such as roads (for safety) and sport fields, and through careful orientation of the light, including the use of shields to reduce light trespass. In particular, illumination of constructions close to water, such as bridges and paths along shores, should be planned so that the illumination of the water is restricted (Hölker et al., 2023). The use of timers or motion detectors can, in turn, limit the temporal distribution of the light (Elgert et al., 2021). Where artificial lighting is intentionally used, such as in aquaculture and light‐assisted fisheries (Ishibashi et al., 2009; Kehayias et al., 2016), management should balance its operational benefits against potential ecological effects on fishes and the surrounding aquatic community.

Light intensities are frequently higher than needed and could be reduced, especially if the illumination is more carefully restricted to the desired area. The spectral tuning of artificial light is enabled by recent advances in LED technology (Longcore et al., 2018). However, species differ in their spectral sensitivity and no specific spectral distribution is optimal for all species (Kivelä et al., 2023; Kivelä et al., 2025; Miller & Rice, 2023; Parkinson & Tiegs, 2023). Yet, avoiding artificial light that mimics daylight is likely to minimise disruptive effects (Owens et al., 2020). However, human discrimination ability decreases with a shift towards longer wavelengths (red light), which needs to be considered in the planning of lighting systems, especially for road safety (Durmus et al., 2024). Red light also requires a higher power input than white light to gain the same light output for human vision (Durmus et al., 2024). Thus, the development of environmentally friendly artificial lighting requires a careful balancing of costs and benefits that considers environmental, social and economic aspects.

Research studies in aquaculture and fisheries are currently providing insights into fish responses to artificial illumination, which can provide valuable mechanistic understanding of the effects of altered light intensity, spectrum and photoperiod on fish biology. Yet this knowledge is underused in assessing the potential ecological consequences of ALAN for wild fishes and should be more widely considered in the development of solutions to the light pollution issue in aquatic ecosystems.

## 11 CONCLUSIONS

1. Aquatic ecosystems are increasingly exposed to artificial light at night (ALAN). Fishes are key components of aquatic ecosystems and the impact of ALAN on them could have major ecological consequences.
2. Our systematic review of the literature reveals that research to date has focused on the direct impacts of ALAN on the behaviour and physiology of fishes, while indirect impacts through species interactions remain largely unexplored. Moreover, the consequences of the responses for population dynamics, species interactions and community composition are poorly understood, and virtually nothing is known about effects on ecosystem functioning or the long‐term ecological and evolutionary consequences.
3. ALAN may interact with other stressors in shaping fish responses, especially with global warming and eutrophication, and more research is needed on these potential interactions.
4. More research should be carried out in the field using experimental designs that capture the complexity of ecosystems. Field research can be complemented by simplified laboratory experiments to disentangle underlying pathways and mechanisms.
5. Light pollution can be reduced relatively easily by adopting better lighting practices, such as targeting light more precisely in space and time and avoiding unnecessarily high intensities. In implementing these measures, social, economic and environmental aspects need to be taken into account.

## AUTHOR CONTRIBUTIONS

Conceptualization: HC and UC. Database review: HC and UC. Writing – original draft: HC. Writing – review & editing: HC and UC. Funding: HC.

## CONFLICT OF INTEREST STATEMENT

The authors declare no conflicts of interest.

## Supporting information


**Table S1.** Number of studies that found or did not find an effect of ALAN on traits of fishes.
**Figure S1**. Logistic regression curves (binomial error distribution, logit link) of the influence of light intensity on the probability of detecting an effect of ALAN on fishes for different response categories. The x‐axis is presented on a log_10_ scale. Lines represent model‐predicted probabilities, and shaded areas indicate 95% confidence intervals around predictions.
**Figure S2**. Number of studies that found (Yes) or did not find (No) an impact of ALAN on fishes depending on the colour of the light used (*N* = 141). NR = not reported.
**Figure S3**. Logistic regression curves (binomial error distribution, logit link) of the influence of ALAN duration on the probability of detecting an effect of ALAN on fishes for different response categories. The x‐axis is presented on a log_10_ scale. Lines represent model‐predicted probabilities, and shaded areas indicate 95% confidence intervals around predictions.

**Data S1.** Appendix S2.

## Data Availability

The data that support the findings of this study are available in the supplementary material of this article.

## References

[jfb70656-bib-0001] Abdelnour, S. A. , Naiel, M. A. E. , Ben Said, M. , Alnajeebi, A. M. , Nasr, F. A. , Al‐Doaiss, A. A. , Mahasneh, Z. M. H. , & Noreldin, A. E. (2024). Environmental epigenetics: Exploring phenotypic plasticity and transgenerational adaptation in fish. Environmental Research, 252, 118799. 10.1016/j.envres.2024.118799 38552831

[jfb70656-bib-0002] Alaasam, V. J. , Hui, C. , Lomas, J. , Ferguson, S. M. , Zhang, Y. , Yim, W. C. , & Ouyang, J. Q. (2024). What happens when the lights are left on? Transcriptomic and phenotypic habituation to light pollution. iScience, 27(2), 108864. 10.1016/j.isci.2024.108864 PMC10839644 38318353

[jfb70656-bib-0004] Bäckroos, S. , Lehtonen, T. K. , & Candolin, U. (2026). Light pollution accelerates development of nuptial colouration in three‐spined stickleback (*Gasterosteus aculeatus*) males. Canadian Journal of Zoology, 104, 1–10. 10.1139/cjz-2026-0032

[jfb70656-bib-0005] Barker, V. A. , & Cowan, J. H. (2018). The effect of artificial light on the community structure of reef‐associated fishes at oil and gas platforms in the northern Gulf of Mexico. Environmental Biology of Fishes, 101(1), 153–166. 10.1007/s10641-017-0688-9

[jfb70656-bib-0006] Bassi, A. , Love, O. P. , Cooke, S. J. , Warriner, T. R. , Harris, C. M. , & Madliger, C. L. (2022). Effects of artificial light at night on fishes: A synthesis with future research priorities. Fish and Fisheries, 23(3), 631–647. 10.1111/faf.12638

[jfb70656-bib-0007] Becker, A. , Whitfield, A. K. , Cowley, P. D. , Jarnegren, J. , & Naesje, T. F. (2013). Potential effects of artificial light associated with anthropogenic infrastructure on the abundance and foraging behaviour of estuary‐associated fishes. Journal of Applied Ecology, 50(1), 43–50. 10.1111/1365-2664.12024

[jfb70656-bib-0008] Berge, J. , Geoffroy, M. , Daase, M. , Cottier, F. , Priou, P. , Cohen, J. H. , Johnsen, G. , McKee, D. , Kostakis, I. , Renaud, P. E. , Vogedes, D. , Anderson, P. , Last, K. S. , & Gauthier, S. (2020). Artificial light during the polar night disrupts Arctic fish and zooplankton behaviour down to 200 m depth. Communications Biology, 3(1), 102. 10.1038/s42003-020-0807-6 PMC7058619 32139805

[jfb70656-bib-0009] Boeuf, G. , & Le Bail, P. Y. (1999). Does light have an influence on fish growth? Aquaculture, 177(1–4), 129–152. 10.1016/s0044-8486(99)00074-5

[jfb70656-bib-0010] Bolton, D. , Mayer‐Pinto, M. , Clark, G. F. , Dafforn, K. A. , Brassil, W. A. , Becker, A. , & Johnston, E. L. (2017). Coastal urban lighting has ecological consequences for multiple trophic levels under the sea. Science of the Total Environment, 576, 1–9. 10.1016/j.scitotenv.2016.10.037 27780095

[jfb70656-bib-0011] Boswell, M. , Lu, Y. , Boswell, W. , Savage, M. , Hildreth, K. , Salinas, R. , Walter, C. A. , & Walter, R. B. (2019). Fluorescent light incites a conserved immune and inflammatory genetic response within vertebrate organs, (Danio rerio Oryzias latipes and Mus musculus). Genes, 10(4), 271. 10.3390/genes10040271 PMC6523474 30987199

[jfb70656-bib-0012] Bradshaw, W. E. , & Holzapfel, C. M. (2010). Light, time, and the physiology of biotic response to rapid climate change in animals. Annual Review of Physiology, 72, 147–166. 10.1146/annurev-physiol-021909-135837 20148671

[jfb70656-bib-0013] Brüning, A. , Hölker, F. , Franke, S. , Preuer, T. , & Kloas, W. (2015). Spotlight on fish: Light pollution affects circadian rhythms of European perch but does not cause stress. Science of the Total Environment, 511, 516–522. 10.1016/j.scitotenv.2014.12.094 25577738

[jfb70656-bib-0014] Brüning, A. , Hölker, F. , & Wolter, C. (2011). Artificial light at night: Implications for early life stages development in four temperate freshwater fish species. Aquatic Sciences, 73(1), 143–152. 10.1007/s00027-010-0167-2

[jfb70656-bib-0015] Brüning, A. , Kloas, W. , Preuer, T. , & Hölker, F. (2018). Influence of artificially induced light pollution on the hormone system of two common fish species, perch and roach, in a rural habitat. Conservation Physiology, 6, coy016. 10.1093/conphys/coy016 PMC5905364 29686874

[jfb70656-bib-0016] Burton, T. , & Metcalfe, N. B. (2014). Can environmental conditions experienced in early life influence future generations? Proceedings of the Royal Society B‐Biological Sciences, 281(1785), 20140311. 10.1098/rspb.2014.0311 PMC4024293 24807254

[jfb70656-bib-0017] Cahill, A. E. , Aiello‐Lammens, M. E. , Fisher‐Reid, M. C. , Hua, X. , Karanewsky, C. J. , Ryu, H. Y. , Sbeglia, G. C. , Spagnolo, F. , Waldron, J. B. , Warsi, O. , & Wiens, J. J. (2013). How does climate change cause extinction? Proceedings of the Royal Society B‐Biological Sciences, 280(1750), 20121890. 10.1098/rspb.2012.1890 PMC3574421 23075836

[jfb70656-bib-0018] Candolin, U. (2019). The threespine stickleback (*Gasterosteus aculeatus*) as a modifier of ecological disturbances. Evolutionary Ecology Research, 20, 167–191.

[jfb70656-bib-0019] Candolin, U. (2024). Coping with light pollution in urban environments: Patterns and challenges. iScience, 27(3), 109244. 10.1016/j.isci.2024.109244 PMC10904992 38433890

[jfb70656-bib-0020] Candolin, U. , & Filippini, T. (2025). Light pollution and its impact on human health and wildlife. BMC Environmental Science, 2(1), 1. 10.1186/s44329-025-00017-7

[jfb70656-bib-0021] Candolin, U. , & Rahman, T. (2023). Behavioural responses of fishes to anthropogenic disturbances: Adaptive value and ecological consequences. Journal of Fish Biology, 103(4), 773–783. 10.1111/jfb.15322 36647916

[jfb70656-bib-0022] Cao, M. , Xu, T. , Song, Y. Q. , Wei, S. , Wang, H. , Guo, X. P. , & Yin, D. Q. (2024). Brominated flame retardant HBCD and artificial light at night Synergically caused visual disorder and sleep difficulty in zebrafish larvae. Environmental Science & Technology, 58(39), 17247–17258. 10.1021/acs.est.4c04342 39291437

[jfb70656-bib-0023] Closs, L. E. , Royan, M. R. , Sayyari, A. , Mayer, I. , Weltzien, F. A. , Baker, D. M. , & Fontaine, R. (2023). Artificial light at night disrupts male dominance relationships and reproductive success in a model fish species. Science of the Total Environment, 900, 166406. 10.1016/j.scitotenv.2023.166406 37597540

[jfb70656-bib-0024] Czarnecka, M. , Kakareko, T. , Jermacz, L. , Pawlak, R. , & Kobak, J. (2019). Combined effects of nocturnal exposure to artificial light and habitat complexity on fish foraging. Science of the Total Environment, 684, 14–22. 10.1016/j.scitotenv.2019.05.280 31150872

[jfb70656-bib-0025] Davies, T. W. , Duff, J. P. , Bennie, J. , & Gaston, K. J. (2014). The nature, extent, and ecological implications of marine light pollution. Frontiers in Ecology and the Environment, 12, 347–355.

[jfb70656-bib-0026] Dominoni, D. M. , Borniger, J. C. , & Nelson, R. J. (2016). Light at night, clocks and health: From humans to wild organisms. Biology Letters, 12(2), 20160015. 10.1098/rsbl.2016.0015 PMC4780560 26888917

[jfb70656-bib-0027] Durmus, D. , Jägerbrand, A. K. , & Tengelin, M. N. (2024). Research note: Red light to mitigate light pollution: Is it possible to balance functionality and ecological impact? Lighting Research & Technology, 56(3), 304–308. 10.1177/14771535231225362

[jfb70656-bib-0028] Elgert, C. , Lehtonen, T. K. , Kaitala, A. , & Candolin, U. (2021). The duration of artificial light defines sexual signalling in the common glow‐worm. Behavioral Ecology and Sociobiology, 75(11), 154. 10.1007/s00265-021-03093-2

[jfb70656-bib-0029] Elvidge, C. D. , Ghosh, T. , Chatterjee, N. , Zhizhin, M. , Sutton, P. C. , & Bazilian, M. (2025). A comprehensive global mapping of offshore lighting. Earth System Science Data, 17(2), 579–594. 10.5194/essd-17-579-2025

[jfb70656-bib-0030] Evans, D. M. (2023). Mitigating the impacts of street lighting on biodiversity and ecosystem functioning. Philosophical Transactions of the Royal Society, B: Biological Sciences, 378(1892), 20220355. 10.1098/rstb.2022.0355 PMC10613540 37899015

[jfb70656-bib-0031] Ferretti, M. , Rossi, F. , Benedetti‐Cecchi, L. , & Maggi, E. (2025). Ecological consequences of artificial light at night on coastal species in natural and artificial habitats: A review. Marine Biology, 172(1), 5. 10.1007/s00227-024-04568-2

[jfb70656-bib-0032] Fobert, E. K. , Schubert, K. P. , & da Silva, K. B. (2021). The influence of spectral composition of artificial light at night on clownfish reproductive success. Journal of Experimental Marine Biology and Ecology, 540, 151559. 10.1016/j.jembe.2021.151559

[jfb70656-bib-0033] Fogg, L. G. , Chung, W. S. , Justin Marshall, N. , Cortesi, F. , & de Busserolles, F. (2023). Multiple rod layers increase the speed and sensitivity of vision in nocturnal reef fishes. Proceedings of the Royal Society B‐Biological Sciences, 290(2011), 20231749. 10.1098/rspb.2023.1749 PMC10688437 37989239

[jfb70656-bib-0034] Foster, J. G. , Algera, D. A. , Brownscombe, J. W. , Zolderdo, A. J. , & Cooke, S. J. (2016). Consequences of different types of Littoral zone light pollution on the parental care behaviour of a freshwater teleost fish. Water, Air, and Soil Pollution, 227(11), 404. 10.1007/s11270-016-3106-6

[jfb70656-bib-0035] Fukunaga, K. , & Takemura, A. (2026). Current knowledge of lunar synchronized spawning in fish. Chronobiology International, 43(2), 156–171. 10.1080/07420528.2025.2591924 41363276

[jfb70656-bib-0036] Ganguly, A. , & Candolin, U. (2023). Impact of light pollution on aquatic invertebrates: Behavioral responses and ecological consequences. Behavioral Ecology and Sociobiology, 77(9), 104. 10.1007/s00265-023-03381-z

[jfb70656-bib-0037] Gaston, K. J. , Davies, T. W. , Nedelec, S. L. , & Holt, L. A. (2017). Impacts of artificial light at night on biological timings. Annual Review of Ecology, Evolution, and Systematics, 48, 49–68. 10.1146/annurev-ecolsys-110316-022745

[jfb70656-bib-0038] Gaston, K. J. , & Sanchez de Miguel, A. (2022). Environmental impacts of artificial light at night. Annual Review of Environment and Resources, 47, 373–398. 10.1146/annurev-environ-112420-014438

[jfb70656-bib-0039] Geoffroy, M. , Langbehn, T. , Priou, P. , Varpe, O. , Johnsen, G. , Le Bris, A. , Fisher, J. A. D. , Daase, M. , McKee, D. , Cohen, J. , & Berge, J. (2021). Pelagic organisms avoid white, blue, and red artificial light from scientific instruments. Scientific Reports, 11(1), 14941. 10.1038/s41598-021-94355-6 PMC8298562 34294780

[jfb70656-bib-0040] Georgiou, D. , Reeves, S. E. , da Silva, K. B. , & Fobert, E. K. (2024). Artificial light at night impacts night‐time activity but not day‐time behaviour in a diurnal coral reef fish. Basic and Applied Ecology, 74, 74–82. 10.1016/j.baae.2023.11.009

[jfb70656-bib-0041] Grubisic, M. , Haim, A. , Bhusal, P. , Dominoni, D. M. , Gabriel, K. M. A. , Jechow, A. , Kupprat, F. , Lerner, A. , Marchant, P. , Riley, W. , Stebelova, K. , van Grunsven, R. H. A. , Zeman, M. , Zubidat, A. E. , & Hölker, F. (2019). Light pollution, circadian photoreception, and melatonin in vertebrates. Sustainability, 11(22), 6400. 10.3390/su11226400

[jfb70656-bib-0042] Haddaway, N. R. , Macura, B. , Whaley, P. , & Pullin, A. S. (2018). ROSES RepOrting standards for systematic evidence syntheses: Pro forma, flow‐diagram and descriptive summary of the plan and conduct of environmental systematic reviews and systematic maps. Environmental Evidence, 7(1), 7. 10.1186/s13750-018-0121-7

[jfb70656-bib-0043] Häder, D. P. , & Gao, K. S. (2023). Aquatic productivity under multiple stressors. Water, 15(4), 817. 10.3390/w15040817

[jfb70656-bib-0044] Halfwerk, W. , & Jerem, P. (2021). A systematic review of research investigating the combined ecological impact of anthropogenic noise and artificial light at night. Frontiers in Ecology and Evolution, 9, 765960. 10.3389/fevo.2021.765950

[jfb70656-bib-0045] Hevroy, E. M. , Boxaspen, K. , Oppedal, F. , Taranger, G. L. , & Holm, J. C. (2003). The effect of artificial light treatment and depth on the infestation of the sea louse *Lepeophtheirus salmonis* on Atlantic salmon (*Salmo salar* L.) culture. Aquaculture, 220(1–4), 1–14. 10.1016/s0044-8486(02)00189-8

[jfb70656-bib-0046] Hirt, M. R. , Evans, D. M. , Miller, C. R. , & Ryser, R. (2023). Light pollution in complex ecological systems. Philosophical Transactions of the Royal Society, B: Biological Sciences, 378(1892), 20220351. 10.1098/rstb.2022.0351 PMC10613538 37899008

[jfb70656-bib-0047] Hölker, F. , Jechow, A. , Schroer, S. , Tockner, K. , & Gessner, M. O. (2023). Light pollution of freshwater ecosystems: Principles, ecological impacts and remedies. Philosophical Transactions of the Royal Society, B: Biological Sciences, 378(1892), 20220360. 10.1098/rstb.2022.0360 PMC10613548 37899012

[jfb70656-bib-0048] Hopkins, G. R. , Gaston, K. J. , Visser, M. E. , Elgar, M. A. , & Jones, T. M. (2018). Artificial light at night as a driver of evolution across urban‐rural landscapes. Frontiers in Ecology and the Environment, 16(8), 472–479. 10.1002/fee.1828

[jfb70656-bib-0049] Ishibashi, Y. , Honryo, T. , Saida, K. , Hagiwara, A. , Miyashita, S. , Sawada, Y. , Okada, T. , & Kurata, M. (2009). Artificial lighting prevents high night‐time mortality of juvenile Pacific bluefin tuna, Thunnus orientalis, caused by poor scotopic vision. Aquaculture, 293(3), 157–163. 10.1016/j.aquaculture.2009.04.029

[jfb70656-bib-0050] Kehayias, G. , Bouliopoulos, D. , Chiotis, N. , & Koutra, P. (2016). A photovoltaic‐battery‐LED lamp raft design for purse seine fishery: Application in a large Mediterranean lake. Fisheries Research, 177, 18–23. 10.1016/j.fishres.2016.01.003

[jfb70656-bib-0051] Kivelä, L. , Elgert, C. , Lehtonen, T. K. , & Candolin, U. (2023). The color of artificial light affects mate attraction in the common glow‐worm. Science of the Total Environment, 857, 159451. 10.1016/j.scitotenv.2022.159451 36252663

[jfb70656-bib-0052] Kivelä, L. , Elgert, C. , Lehtonen, T. K. , & Candolin, U. (2025). Mitigating the light pollution problem via spectral adjustment: Color‐biased phototaxis in male glow‐worms. Oecologia, 207(8), 133. 10.1007/s00442-025-05768-3 PMC12255557 40650820

[jfb70656-bib-0053] Kloas, W. , Stöck, M. , Lutz, I. , & Ziková‐Kloas, A. (2024). Endocrine disruption in teleosts and amphibians is mediated by anthropogenic and natural environmental factors: Implications for risk assessment. Philosophical Transactions of the Royal Society, B: Biological Sciences, 379(1898), 20220505. 10.1098/rstb.2022.0505 PMC10838649 38310939

[jfb70656-bib-0054] Kupprat, F. , Hölker, F. , & Kloas, W. (2020). Can skyglow reduce nocturnal melatonin concentrations in Eurasian perch? Environmental Pollution, 262, 114324. 10.1016/j.envpol.2020.114324 32179225

[jfb70656-bib-0055] Kupprat, F. , Kloas, W. , Krüger, A. , Schmalsch, C. , & Hölker, F. (2021). Misbalance of thyroid hormones after two weeks of exposure to artificial light at night in Eurasian perch *Perca fluviatilis* . Conservation Physiology, 9, coaa124. 10.1093/conphys/coaa124 PMC7905158 33659060

[jfb70656-bib-0056] Kurvers, R. , Drägestein, J. , Hölker, F. , Jechow, A. , Krause, J. , & Bierbach, D. (2018). Artificial light at night affects emergence from a refuge and space use in guppies. Scientific Reports, 8, 14131. 10.1038/s41598-018-32466-3 PMC6147999 30237438

[jfb70656-bib-0057] Kyba, C. , Mohar, A. , & Posch, T. (2017). How bright is moonlight? Astronomy & Geophysics, 58(1), 1.31–31.32. 10.1093/astrogeo/atx025

[jfb70656-bib-0058] Kyba, C. C. M. , Altintas, Y. O. , Walker, C. E. , & Newhouse, M. (2023). Citizen scientists report global rapid reductions in the visibility of stars from 2011 to 2022. Science, 379(6629), 265–268. 10.1126/science.abq7781 36656932

[jfb70656-bib-0059] Lagisz, M. , Yang, Y. F. , Young, S. , & Nakagawa, S. (2025). A practical guide to evaluating sensitivity of literature search strings for systematic reviews using relative recall. Research Synthesis Methods, 16(1), 1–14. 10.1017/rsm.2024.6 PMC12621535 41626904

[jfb70656-bib-0060] Le Roy, A. , & Seebacher, F. (2020). Mismatched light and temperature cues disrupt locomotion and energetics via thyroid‐dependent mechanisms. *Conservation* . Physiology, 8, coaa051. 10.1093/conphys/coaa051 PMC7287392 32547766

[jfb70656-bib-0061] Lehtonen, A. , & Candolin, U. (2025). Light pollution suppresses diel vertical migration across rural and urban *Daphnia magna* populations. BMC Environmental Science, 2, 22. 10.1186/s44329-025-00036-4

[jfb70656-bib-0062] Li, W. W. , Zhang, D. X. , Zou, Q. Q. , Bose, A. P. H. , Jordan, A. , McCallum, E. S. , Bao, J. H. , & Duan, M. (2024). Behavioural and transgenerational effects of artificial light at night (ALAN) of varying spectral compositions in zebrafish (*Danio rerio*). Science of the Total Environment, 954, 176336. 10.1016/j.scitotenv.2024.176336 39299330

[jfb70656-bib-0063] Lin, C. Y. , Dai, H. C. , Shi, X. T. , Deng, Z. D. , Mao, J. Q. , Luo, J. , Huang, W. Q. , Xu, J. W. , Zhang, N. , & Sun, S. K. (2021). Investigating feasible light configurations for fish restoration: An ethological insight. Fisheries Research, 234, 105807. 10.1016/j.fishres.2020.105807

[jfb70656-bib-0064] Liu, J. A. , Meléndez‐Fernández, O. H. , Bumgarner, J. R. , & Nelson, R. J. (2022). Effects of light pollution on photoperiod‐driven seasonality. Hormones and Behavior, 141, 105150. 10.1016/j.yhbeh.2022.105150 PMC10137835 35304351

[jfb70656-bib-0065] Longcore, T. , Rodriguez, A. , Witherington, B. , Penniman, J. F. , Herf, L. , & Herf, M. (2018). Rapid assessment of lamp spectrum to quantify ecological effects of light at night [article]. Journal of Experimental Zoology Part A‐Ecological and Integrative Physiology, 329(8–9), 511–521. 10.1002/jez.2184 29894022

[jfb70656-bib-0066] Lucon‐Xiccato, T. , De Russi, G. , Cannicci, S. , Maggi, E. , & Bertolucci, C. (2023). Embryonic exposure to artificial light at night impairs learning abilities and their covariance with behavioural traits in teleost fish. Biology Letters, 19(11), 20230436. 10.1098/rsbl.2023.0436 PMC10663786 37990566

[jfb70656-bib-0067] Ma, H. Y. , Tan, M. J. , Li, W. W. , Xu, Q. Y. , & Duan, M. (2026). Effects of different LED lights colors on the feeding behavior of Chinese longsnout catfish (Leiocassis longirostris). Aquaculture, 612, 743124. 10.1016/j.aquaculture.2025.743124

[jfb70656-bib-0068] MacAulay, S. , & Cable, J. (2024). *Gyrodactylus* in the spotlight: How exposure to light impacts disease and the feeding behavior of the freshwater tropical guppy (*Poecilia reticulata*). Journal of Fish Biology, 105(3), 682–690. 10.1111/jfb.15816 38828698

[jfb70656-bib-0069] Maggi, E. , Bertocci, I. , & Benedetti‐Cecchi, L. (2020). Light pollution enhances temporal variability of photosynthetic activity in mature and developing biofilm. Hydrobiologia, 847(7), 1793–1802. 10.1007/s10750-019-04102-2

[jfb70656-bib-0070] Manfrin, A. , Singer, G. , Larsen, S. , Weiss, N. , van Grunsven, R. H. A. , Weiss, N. S. , Wohlfahrt, S. , Monaghan, M. T. , & Holker, F. (2017). Artificial light at night affects organism flux across ecosystem boundaries and drives community structure in the recipient ecosystem. Frontiers in Environmental Science, 5, 61. 10.3389/fenvs.2017.00061

[jfb70656-bib-0071] Marangoni, L. F. B. , Davies, T. , Smyth, T. , Rodríguez, A. , Hamann, M. , Duarte, C. , Pendoley, K. , Berge, J. , Maggi, E. , & Levy, O. (2022). Impacts of artificial light at night in marine ecosystems‐a review. Global Change Biology, 28(18), 5346–5367. 10.1111/gcb.16264 PMC9540822 35583661

[jfb70656-bib-0072] Marshall, N. J. , Powell, S. B. , Cronin, T. W. , Caldwell, R. L. , Johnsen, S. , Gruev, V. , Chiou, T. H. S. , Roberts, N. W. , & How, M. J. (2019). Polarisation signals: A new currency for communication. Journal of Experimental Biology, 222(3), jeb134213. 10.1242/jeb.134213 30733259

[jfb70656-bib-0073] Martins, L. P. , Garcia‐Callejas, D. , Lai, H. R. , Wootton, K. L. , & Tylianakis, J. M. (2024). The propagation of disturbances in ecological networks. Trends in Ecology & Evolution, 39(6), 558–570. 10.1016/j.tree.2024.01.009 38402007

[jfb70656-bib-0074] Miller, C. R. , & Rice, A. N. (2023). A synthesis of the risks of marine light pollution across organismal and ecological scales. Aquatic Conservation: Marine and Freshwater Ecosystems, 33(12), 1590–1602. 10.1002/aqc.4011

[jfb70656-bib-0075] Mobley, C. D. (1994). Light and water, radiative transfer in natural waters. Academic Press.

[jfb70656-bib-0076] Munson, A. , Cortese, D. , & Killen, S. S. (2024). Multiple stressors. In B. B. M. Wong & U. Candolin (Eds.), Behavioural responses to a changing world: Challenges and applications (pp. 171–187). Oxford University Press.

[jfb70656-bib-0077] Musilova, Z. , Salzburger, W. , & Cortesi, F. (2021). The visual opsin gene repertoires of teleost fishes: Evolution, ecology, and function. Annual Review of Cell and Developmental Biology, 37(1), 441–468. 10.1146/annurev-cellbio-120219-024915 34351785

[jfb70656-bib-0078] Nelson, T. R. , Michel, C. J. , Gary, M. P. , Lehman, B. M. , Demetras, N. J. , Dudley, P. N. , Hammen, J. J. , & Horn, M. J. (2022). Riverine fish density, predator‐prey interactions, and their relationships with artificial light at night. Ecosphere, 13(10), e4261. 10.1002/ecs2.4261

[jfb70656-bib-0079] Newman, R. C. , Ellis, T. , Davison, P. I. , Ives, M. J. , Thomas, R. J. , Griffiths, S. W. , & Riley, W. D. (2015). Using novel methodologies to examine the impact of artificial light at night on the cortisol stress response in dispersing Atlantic salmon (*Salmo salar* L.) fry. Conservation Physiology, 3, cov051. 10.1093/conphys/cov051 PMC4778446 27293735

[jfb70656-bib-0080] Norberg, B. , Brown, C. L. , Halldorsson, A. , Stensland, K. , & Björnsson, B. T. (2004). Photoperiod regulates the timing of sexual maturation, spawning, sex steroid and thyroid hormone profiles in the Atlantic cod (*Gadus morhua*). Aquaculture, 229(1–4), 451–467. 10.1016/s0044-8486(03)00393-4

[jfb70656-bib-0081] O'Connor, J. J. , Fobert, E. K. , Besson, M. , Jacob, H. , & Lecchini, D. (2019). Live fast, die young: Behavioural and physiological impacts of light pollution on a marine fish during larval recruitment. Marine Pollution Bulletin, 146, 908–914. 10.1016/j.marpolbul.2019.05.038 31426235

[jfb70656-bib-0082] Owens, A. C. S. , Cochard, P. , Durrant, J. , Farnworth, B. , Perkin, E. K. , & Seymoure, B. (2020). Light pollution is a driver of insect declines [article]. Biological Conservation, 241, 108259. 10.1016/j.biocon.2019.108259

[jfb70656-bib-0083] Parkinson, E. , & Tiegs, S. D. (2023). Spectral composition of light‐emitting diodes impacts aquatic and terrestrial invertebrate communities with potential implications for cross‐ecosystem subsidies. Philosophical Transactions of the Royal Society, B: Biological Sciences, 378(1892), 20220361. 10.1098/rstb.2022.0361 PMC10613537 37899013

[jfb70656-bib-0084] Perkin, E. K. , Holker, F. , Tockner, K. , & Richardson, J. S. (2014). Artificial light as a disturbance to light‐naive streams. Freshwater Biology, 59(11), 2235–2244. 10.1111/fwb.12426

[jfb70656-bib-0085] Pulgar, J. , Manríquez, P. H. , Widdicombe, S. , Garcia‐Huidobro, R. , Quijón, P. A. , Carter, M. , Aldana, M. , Quintanilla‐Ahumada, D. , & Duarte, C. (2023). Artificial light at night (ALAN) causes size‐dependent effects on intertidal fish decision‐making. Marine Pollution Bulletin, 193, 115190. 10.1016/j.marpolbul.2023.115190 37336043

[jfb70656-bib-0086] Pulgar, J. , Zeballos, D. , Vargas, J. , Aldana, M. , Manriquez, P. H. , Manriquez, K. , Quijón, P. A. , Widdicombe, S. , Anguita, C. , Quintanilla, D. , & Duarte, C. (2019). Endogenous cycles, activity patterns and energy expenditure of an intertidal fish is modified by artificial light pollution at night (ALAN). Environmental Pollution, 244, 361–366. 10.1016/j.envpol.2018.10.063 30352350

[jfb70656-bib-0087] Rahman, T. , & Candolin, U. (2022). Linking animal behavior to ecosystem change in disturbed environments. Frontiers in Ecology and Evolution, 10, 893453. 10.3389/fevo.2022.893453

[jfb70656-bib-0088] Rennison, D. J. , Owens, G. L. , Heckman, N. , Schluter, D. , & Veen, T. (2016). Rapid adaptive evolution of colour vision in the threespine stickleback radiation. Proceedings of the Royal Society B‐Biological Sciences, 283(1830), 20160242. 10.1098/rspb.2016.0242 PMC4874711 27147098

[jfb70656-bib-0089] Riley, W. D. , Davison, P. I. , Maxwell, D. L. , Newman, R. C. , & Ives, M. J. (2015). A laboratory experiment to determine the dispersal response of Atlantic salmon (*Salmo salar*) fry to street light intensity. Freshwater Biology, 60(5), 1016–1028. 10.1111/fwb.12568

[jfb70656-bib-0090] Roost, T. , Hargous, J. , Van Espen, L. , Schligler, J. , Killen, S. S. , Beldade, R. , Swearer, S. E. , & Mills, S. C. (2025). Artificial light at night during early development directly affects embryonic but not larval quality in a wild coral reef fish. Conservation Physiology, 13(1), coaf041. 10.1093/conphys/coaf041 PMC12203906 40584600

[jfb70656-bib-0091] Sanders, D. , Frago, E. , Kehoe, R. , Patterson, C. , & Gaston, K. J. (2021). A meta‐analysis of biological impacts of artificial light at night. Nature Ecology & Evolution, 5(1), 74–81. 10.1038/s41559-020-01322-x 33139919

[jfb70656-bib-0092] Schligler, J. , Cortese, D. , Beldade, R. , Swearer, S. E. , & Mills, S. C. (2021). Long‐term exposure to artificial light at night in the wild decreases survival and growth of a coral reef fish. Proceedings of the Royal Society B‐Biological Sciences, 288(1952), 20210454. 10.1098/rspb.2021.0454 PMC8187998 34102892

[jfb70656-bib-0093] Schligler, J. , Norin, T. , McBride, M. , Morat, F. , Killen, S. S. , Swearer, S. E. , Beldade, R. , & Mills, S. C. (2026). Fatal attraction: Light pollution creates an ecological trap for wild fish. Global Change Biology, 32(1), e70645. 10.1111/gcb.70645 41486759

[jfb70656-bib-0094] Shafiei‐Sabet, S. , Van Dooren, D. , & Slabbekoorn, H. (2016). Son et lumiere: Sound and light effects on spatial distribution and swimming behavior in captive zebrafish. Environmental Pollution, 212, 480–488. 10.1016/j.envpol.2016.02.046 26963699

[jfb70656-bib-0095] Sichrowsky, U. , Schabetsberger, R. , Gassner, H. , Kaiser, R. , Boufana, B. , & Psenner, R. (2013). Cradle or plague pit? Illuminated cages increase the transmission risk of parasites from copepods to coregonids. Aquaculture, 392, 8–15. 10.1016/j.aquaculture.2013.01.031

[jfb70656-bib-0096] Smyth, T. J. , Wright, A. E. , Edwards‐Jones, A. , McKee, D. , Queirós, A. , Rendon, O. , Tidau, S. , & Davies, T. W. (2022). Disruption of marine habitats by artificial light at night from global coastal megacities. Elementa: Science of the Anthropocene, 10(1), 00042. 10.1525/elementa.2022.00042

[jfb70656-bib-0097] Szekeres, P. , Wilson, A. D. , Haak, C. R. , Danylchuk, A. J. , Brownscombe, J. W. , Elvidge, C. K. , Shultz, A. D. , Birnie‐Gauvin, K. , & Cooke, S. J. (2017). Does coastal light pollution alter the nocturnal behavior and blood physiology of juvenile bonefish (*Albula vulpes*)? Bulletin of Marine Science, 93(2), 491–505. 10.5343/bms.2016.1061

[jfb70656-bib-0098] Tabor, R. A. , Perkin, E. K. , Beauchamp, D. A. , Britt, L. L. , Haehn, R. , Green, J. , Robinson, T. , Stolnack, S. , Lantz, D. W. , & Moore, Z. J. (2021). Artificial lights with different spectra do not alter detrimental attraction of young Chinook salmon and sockeye salmon along lake shorelines. Lake and Reservoir Management, 37(3), 313–322. 10.1080/10402381.2021.1906364

[jfb70656-bib-0099] Tałanda, J. , Maszczyk, P. , Babkiewicz, E. , Rutkowska, K. , & Slusarczyk, M. (2022). The short‐term effects of planktivorous fish foraging in the presence of artificial light at night on lake zooplankton. Journal of Plankton Research, 44(6), 954–958. 10.1093/plankt/fbac046 PMC9692195 36447780

[jfb70656-bib-0100] Thoré, E. S. J. , Aulsebrook, A. E. , Brand, J. A. , Almeida, R. A. , Brodin, T. , & Bertram, M. G. (2024). Time is of the essence: The importance of considering biological rhythms in an increasingly polluted world. PLoS Biology, 22(11), e3002478. 10.1371/journal.pbio.3002478 PMC10826942 38289905

[jfb70656-bib-0101] Tidau, S. , Smyth, T. , McKee, D. , Wiedenmann, J. , D'Angelo, C. , Wilcockson, D. , Ellison, A. , Grimmer, A. J. , Jenkins, S. R. , Widdicombe, S. , Queiros, A. M. , Talbot, E. , Wright, A. , & Davies, T. W. (2021). Marine artificial light at night: An empirical and technical guide. Methods in Ecology and Evolution, 12(9), 1588–1601. 10.1111/2041-210x.13653

[jfb70656-bib-0102] Tsounis, L. , & Kehayias, G. (2021). Alteration of the feeding behavior of an omnivorous fish, *Scardinius acarnanicus* (Actinopterygii: Cypriniformes: Cyprinidae), in the presence of fishing lights. Acta Ichthyologica et Piscatoria, 51(2), 131–138. 10.3897/aiep.51.63299

[jfb70656-bib-0103] Tuomainen, U. , & Candolin, U. (2011). Behavioural responses to human‐induced environmental change [article]. Biological Reviews, 86(3), 640–657. 10.1111/j.1469-185X.2010.00164.x 20977599

[jfb70656-bib-0104] Villéger, S. , Brosse, S. , Mouchet, M. , Mouillot, D. , & Vanni, M. J. (2017). Functional ecology of fish: Current approaches and future challenges. Aquatic Sciences, 79(4), 783–801. 10.1007/s00027-017-0546-z

[jfb70656-bib-0105] Weschke, E. , Schligler, J. , Hely, I. , Roost, T. , Schies, J. A. , Williams, B. , Dworzanski, B. , Mills, S. C. , Beldade, R. , Simpson, S. D. , & Radford, A. N. (2024). Artificial light increases nighttime prevalence of predatory fishes, altering community composition on coral reefs. Global Change Biology, 30(12), e70002. 10.1111/gcb.70002 PMC11653166 39692005

[jfb70656-bib-0106] Wong, B. B. M. , & Candolin, U. (2015). Behavioral responses to changing environments [review]. Behavioral Ecology, 26(3), 665–673. 10.1093/beheco/aru183

[jfb70656-bib-0107] Zapata, M. J. , Sullivan, S. M. P. , & Gray, S. M. (2019). Artificial lighting at night in estuaries: Implications from individuals to ecosystems. Estuaries and Coasts, 42(2), 309–330. 10.1007/s12237-018-0479-3

[jfb70656-bib-0108] Zhang, G. B. , Ye, Z. Q. , Jiang, Z. J. , Wu, C. Q. , Ge, L. F. , Wang, J. X. , Xu, X. W. , Wang, T. M. , & Yang, J. W. (2024). Circadian patterns and photoperiodic modulation of clock gene expression and neuroendocrine hormone secretion in the marine teleost *Larimichthys crocea* . Chronobiology International, 41(3), 329–346. 10.1080/07420528.2024.2315215 38516993

