# Supplementary material for: Impact of light pollution on fishes: Mechanisms and ecological consequences
Source: J Fish Biol. 2026 Sep 17;109(5):2538–52. doi: 10.1111/jfb.70656 (PMC13770688; doi:10.1111/jfb.70656)
Supplement: Supplementary file 1 [file JFB-109-2538-s002.docx]

Supplementary materials to:

**Impact of light pollution on fishes: mechanisms and ecological consequences**

Hanxi Chen and Ulrika Candolin

**TABLE S1.** Number of studies that found or did not find an effect of ALAN on traits of fishes.

| **Impact** | | **Number of studies** |
| --- | --- | --- |
| **Yes** |  | **86** |
|  | Abundance | 7 |
|  | Behaviour | 40 |
|  | Cognition | 3 |
|  | Distribution | 1 |
|  | Gene expression | 3 |
|  | Growth | 3 |
|  | Physiology | 22 |
|  | Reproduction | 2 |
|  | Survival | 5 |
| **No** |  | **55** |
|  | Abundance | 1 |
|  | Behaviour | 22 |
|  | Cognition | 2 |
|  | Distribution | 0 |
|  | Gene expression | 2 |
|  | Growth | 3 |
|  | Physiology | 16 |
|  | Reproduction | 5 |
|  | Survival | 4 |
| **Total** |  | **141** |

**FIGURE S1** Logistic regression curves (binomial error distribution, logit link) of the influence of light intensity on the probability of detecting an effect of ALAN on fishes for different response categories. The x-axis is presented on a log₁₀ scale. Lines represent model-predicted probabilities, and shaded areas indicate 95% confidence intervals around predictions.

**FIGURE S2** Number of studies that found (Yes) or did not find (No) an impact of ALAN on fishes depending on the colour of the light used (N = 141). NR = not reported.

**FIGURE S3** Logistic regression curves (binomial error distribution, logit link) of the influence of ALAN duration on the probability of detecting an effect of ALAN on fishes for different response categories. The x-axis is presented on a log₁₀ scale. Lines represent model-predicted probabilities, and shaded areas indicate 95% confidence intervals around predictions.
